# Early and long-standing rheumatoid arthritis: distinct molecular signatures identified by gene-expression profiling in synovia

**DOI:** 10.1186/ar2744

**Published:** 2009-06-29

**Authors:** Thierry Lequerré, Carine Bansard, Olivier Vittecoq, Céline Derambure, Martine Hiron, Maryvonne Daveau, François Tron, Xavier Ayral, Norman Biga, Isabelle Auquit-Auckbur, Gilles Chiocchia, Xavier Le Loët, Jean-Philippe Salier

**Affiliations:** 1 Department of Rheumatology, Rouen University Hospital and Inserm 905 & Institut Fédératif de Recherche Multidisciplinaire sur les Peptides 23, Institute for Biomedical Research, University of Rouen, 76031 Rouen Cedex, and Consortium EGERIE, France; 2 Inserm Unité 905, Institut Fédératif de Recherche Multidisciplinaire sur les Peptides 23, Institute for Biomedical Research, University of Rouen, Faculté de Médecine-Pharmacie, Rouen, France; 3 Department of Rheumatology, Cochin Hospital, AP-HP, University of Paris-Descartes, 27, rue du faubourg Saint-Jacques, 75679 Paris Cedex 14, France; 4 Department of Orthopedic and Traumatology, Rouen University Hospital, 76031 Rouen Cedex, France; 5 Department of Plastic and Reconstructive Surgery, Rouen University Hospital, 76031 Rouen Cedex, France; 6 Institut Cochin, Université Paris Descartes, Inserm (U567), CNRS (UMR8104), Paris, and Consortium EGERIE, France

## Abstract

**Introduction:**

Rheumatoid arthritis (RA) is a heterogeneous disease and its underlying molecular mechanisms are still poorly understood. Because previous microarray studies have only focused on long-standing (LS) RA compared to osteoarthritis, we aimed to compare the molecular profiles of early and LS RA versus control synovia.

**Methods:**

Synovial biopsies were obtained by arthroscopy from 15 patients (4 early untreated RA, 4 treated LS RA and 7 controls, who had traumatic or mechanical lesions). Extracted mRNAs were used for large-scale gene-expression profiling. The different gene-expression combinations identified by comparison of profiles of early, LS RA and healthy synovia were linked to the biological processes involved in each situation.

**Results:**

Three combinations of 719, 116 and 52 transcripts discriminated, respectively, early from LS RA, and early or LS RA from healthy synovia. We identified several gene clusters and distinct molecular signatures specifically expressed during early or LS RA, thereby suggesting the involvement of different pathophysiological mechanisms during the course of RA.

**Conclusions:**

Early and LS RA have distinct molecular signatures with different biological processes participating at different times during the course of the disease. These results suggest that better knowledge of the main biological processes involved at a given RA stage might help to choose the most appropriate treatment.

## Introduction

Rheumatoid arthritis (RA) is a chronic, autoimmune, and inflammatory polyarthritis that induces joint damage and disability. It is a heterogeneous disease with different clinical presentations and courses, ranging from mild to severe. Histological and molecular variations in synovial tissues were previously described among RA patients [1,2]. However, the few microarray studies available were conducted on human RA synovia from patients with long-standing (LS) disease and/or treated with disease-modifying antirheumatic drugs (DMARDs) and/or glucocorticoids [2-4]. Moreover, many studies used osteoarthritis synovia as the control because of the difficulties in obtaining healthy synovial samples [5-8]. The molecular differences observed in those comparisons did not precisely indicate the pathological processes involved in RA, especially if we consider that different biological processes are at work throughout the course of RA. The tight link between the molecular pattern and the disease stage was previously described in murine autoimmune arthritis but no data are available on human RA [9]. Therefore, we applied gene-expression profiling to synovial biopsies from patients with early untreated or treated LS RA and control synovia to try to identify biological processes corresponding to the RA stages.

## Materials and methods

### Patients

Synovial tissue biopsies were obtained by arthroscopy from four untreated patients with early RA, four treated patients with LS RA, and seven patients undergoing knee arthroscopy for traumatic or mechanical ligament or meniscus lesions. This protocol (00/149 HP) was approved by our regional ethics committee (CPP Nord-Ouest 1, formerly CCPPRB Haute-Normandie, France), and all participants gave their written informed consent. Clinical and demographic data for these 15 patients are summarized in Table S1 of Additional data file 1. Three of the four early RA patients had rheumatoid factors and/or anti-cyclic citrullinated peptide (n = 1) autoantibodies or structural damage (n = 2) (that is, the main RA characteristics). All early RA patients were untreated (that is, they had not yet been prescribed DMARDs or glucocorticoids), whereas all LS RA patients were taking oral methotrexate. All biopsies were taken from inflamed synovial sites, with at least three samples being taken and pooled to minimize the heterogeneity of cellular infiltrates and fibrosis among them.

### Sample preparation and cDNA arrays

A standard phenol-chloroform procedure was used to extract total RNAs from synovial tissue, and quality was controlled on an Agilent 2100 Bioanalyzer (Agilent Technologies, Inc., Santa Clara, CA, USA). mRNAs were amplified linearly using the MessageAmp™ aRNA [antisense RNA] Kit (Ambion, now part of Applied Biosystems, Huntingdon, UK) in accordance with the instructions of the manufacturer. aRNAs were labeled with [α-^33^P]dCTP. The resulting labeled cDNAs were immediately used for hybridization on an array covering about 10,000 nonredundant genes, as described and validated previously [10,11]. Each RNA sample was hybridized three times on separate arrays.

### Image analysis and data mining

The scanned 16-bit image was imported into a Linux workstation and the spots were automatically identified and analyzed with XDotsReader software, version 1.8 (COSE, Dugny, France) [10]. Spots were identified with the 'algorithm 2' option (that is, spot morphology is the critical parameter), and every spot within an array was quantitated with the 'weighted mean intensity' option (that is, the average of the pixel intensities of the spot is weighted by the distance to the center of this spot). Because of the background homogeneity in our array, a global background over the entire image was calculated as the mean of the local background measured around each spot ('global background' option) and it was automatically subtracted from every spot signal. Next, the mean signal associated with negative control spots was calculated over the entire image. For all genes considered, a spot whose signal was below a given threshold (2 standard deviations above the mean signal for negative controls) was considered to be nonsignificant (*P *< 0.05). Finally, the mean signal associated with negative control spots was automatically subtracted from the signal of every significant spot. To be able to compare images, the mean of the signals provided by the complete set of spots per image served as the basis for normalization.

A transcript was considered to be expressed when at least two hybridizations provided a positive signal. The TIGR MultiExperiment Viewer [12] was used for unsupervised hierarchical clustering by applying the average dot product and complete linkage options, and the supervised statistical tools one-class *t *test with the adjusted Bonferroni correction was used for identification of discriminant transcripts. Adjusted Bonferroni *P *values of less than 0.01 were considered significant.

To determine which biological processes were represented by the differences in gene-expression levels for each comparison (early versus LS RA; early RA or LS RA versus controls), we applied gene-ontology analysis using the PANTHER (Protein ANalysis THrough Evolutionary Relationships) database [13-15]. Genes with eligible symbols available and being categorized by gene-ontology terms were retained. We used the PANTHER binomial statistics tool to compare classifications of multiple clusters of gene lists with a reference list (list of all genes in our array) and to statistically determine over- or underrepresentation of PANTHER classification categories. Each list was compared with the reference list, using the binomial test for each molecular function, biological process, or pathway term in PANTHER. A *P *value was determined using the binomial statistic (that is, the probability that the number of genes observed in each category occurred by chance [randomly], as determined by the reference list). We selected a cutoff of 0.05 to ensure the significance of biological processes and pathways within each ontology term. Compilation of our clinical and experimental data complied with the recommendations for Minimum Information About a Microarray Experiment (MIAME), and the raw data have been deposited [GEO: GSE13026] in the Gene Expression Omnibus (GEO) repository [16].

## Results

### Transcriptome analysis of early versus long-standing rheumatoid arthritis

**Figure 1:**
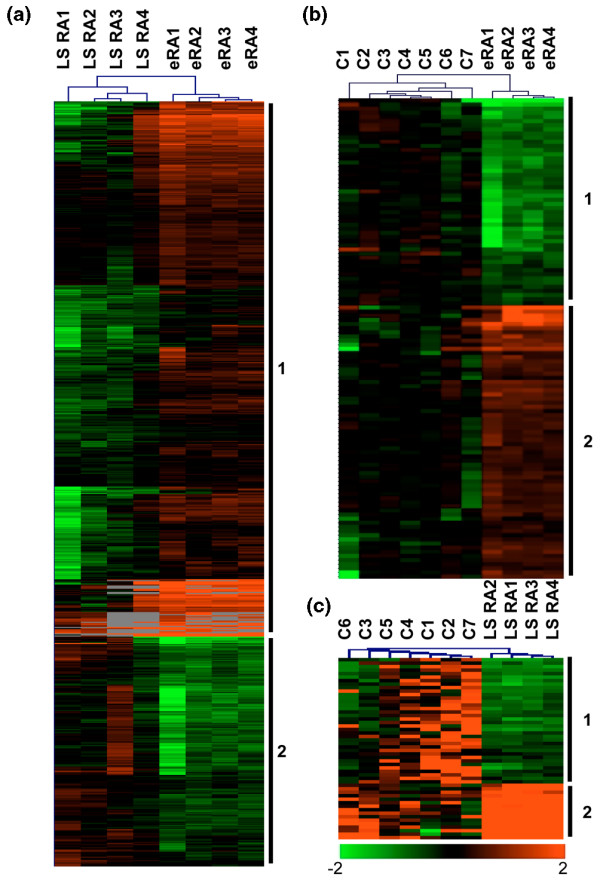
Hierarchical gene clustering in rheumatoid arthritis (RA) and control synovia at different stages of progression. Samples from four early RA (eRA) patients, four long-standing RA (LS RA) patients, and seven controls (C) were studied by microarray analysis. Transcripts were selected with a supervised statistical one-class *t *test with the adjusted Bonferroni correction (*P *< 0.01). Patients are indicated as vertical column headings, and gene symbols of transcripts are given in horizontal rows. Transcript levels are expressed as the ratio sample level/median control level. Scale bar (log_2 _ratio): low (green), high (red), or ratio of 1 (black) expression in the sample versus the median control value (gray squares are missing values). **(a) **Visualization of 719 transcripts able to distinguish LS RA from early RA patients. **(b) **Visualization of 116 transcripts able to distinguish early RA patients from controls. **(c) **Visualization of 52 transcripts able to distinguish LS RA patients from controls. Each clustering is divided in two, yielding clusters 1 and 2, indicated by vertical bars.

Using a *t *test with an adjusted Bonferroni *P *value of less than 0.01, we identified a combination of 719 genes differentially regulated between early and LS RA samples (Figure 1a) (Table S2 in Additional data file 2). These genes clearly separated early and LS RA. Among these 719 genes, 2 clusters (1 and 2) were distinct by their opposite gene-expression regulations for each stage. In cluster 1, 503 genes were more strongly expressed in early than LS RA. Based on gene ontology, we subsequently identified 12 significant (*P *< 0.05) biological processes, including immunity and host defenses, stress responses, T cell-mediated immunity, and tumor suppressor and major histocompatibility complex (MHC) class II-mediated immunity, whose corresponding genes were significantly upregulated during early RA (Table S3 in Additional data file 3). Many pathways involved in this gene cluster were also significantly recognized (*P *< 0.05): T-cell activation, endothelin signaling pathway, hypoxia response, and plasminogen-activating cascade. In cluster 2, 216 genes were expressed significantly more in LS than early RA. The main biological processes implicated in this cluster were cell cycle, cell surface receptor-mediated signal transduction, cell cycle control, ligand-mediated signaling, apoptosis inhibition, and granulocyte-mediated immunity (Table S3 in Additional data file 3). Overall, these findings showed that early and LS RA are distinguishable by their own molecular patterns. We observed that some genes upregulated during early RA were more specifically involved in stress responses, defense mechanisms, and apoptotic processes, whereas upregulated genes in LS RA participated in proliferative processes.

### Transcriptome analysis of early rheumatoid arthritis versus control synovia

To identify molecular mechanisms particularly involved in early RA, we compared the gene-expression levels between early RA and healthy synovia. These samples, too, clearly separated into two clusters in early RA samples: cluster 1 contained 116 genes, 50 of which were downregulated, and cluster 2, consisted of 66 upregulated genes (Figure 1b) (Table S4 in Additional data file 4). Cluster 1 significantly (*P *< 0.05) defined 6 biological processes, 5 predominant molecular functions, and 1 pathway, whereas the 66 genes in cluster 2 clearly involved 6 biological processes, 7 main molecular functions, and 2 pathways (Table 1).

**Table 1:** Ontological classes of differentially expressed genes in early rheumatoid arthritis versus controls

	Gene regulation	Early rheumatoid arthritis versus controls	*P *value
Biological processes			
	Up (cluster 2)		
		Reverse transcription	0.007
		Amino acid activation	0.01
		Protein biosynthesis^a^	0.02
		Nucleoside, nucleotide, and nucleic acid metabolism^a^	0.02
		Protein metabolism and modification	0.03
		Other immune and defense	0.04
	Down (cluster 1)		
		Granulocyte-mediated immunity	0.0004
		Other cell cycle process	0.01
		Macrophage-mediated immunity	0.02
		T cell-mediated immunity	0.03
		Other blood circulation and gas exchange activity	0.04
		Mitochondrial transport	0.05
Molecular functions			
	Up (cluster 2)		
		Ribosomal protein^a^	0.005
		Reverse transcriptase	0.007
		Nucleic acid binding	0.009
		Other transcription factor	0.03
		Defense/immunity protein	0.03
		Nuclease	0.04
		Antibacterial response protein	0.05
	Down (cluster 1)		
		Transmembrane receptor regulatory/adaptor protein	0.008
		Metalloprotease inhibitor^a^	0.02
		Phosphorylase	0.03
		Protease inhibitor	0.03
		Cysteine protease inhibitor	0.04
Pathways			
	Up (cluster 2)		
		Bupropion degradation	0.007
		Salvage pyrimidine deoxyribonucleotides^a^	0.01
	Down (cluster 1)		
		Metabotropic glutamide receptor group I pathway	0.07

The gene-ontology terms used are those of the PANTHER (Protein ANalysis THrough Evolutionary Relationships) database. ^a^Common ontological classes found in both comparisons: early rheumatoid arthritis (RA) or long-standing RA versus controls (see also Table 2).

### Transcriptome analysis of long-standing rheumatoid arthritis versus control synovia

To identify the different biological processes represented in LS RA as opposed to early RA, we compared LS RA and healthy synovia. As shown in Figure 1c, 52 transcripts were able to clearly discriminate between them (Table S5 in Additional data file 5). Among them, 36 (cluster 1) and 16 (cluster 2) genes, respectively, were significantly down- and upregulated in LS RA compared with healthy synovia. Seven and three biological processes and six and five different molecular functions were identified in clusters 1 and 2, respectively, along with three different pathways in each cluster (Table 2). Overall, most of the ontological classes differed between early and LS RA and therefore could be considered specific to the disease stage. However, a few overlaps with opposite regulations were observed. Indeed, regardless of the RA stage, genes coding for metalloprotease inhibitors were downregulated and those coding for the salvage pyrimidine deoxyribonucleotide pathway were upregulated. These ontological classes seemed to be disease-specific. Conversely, the genes of some biological processes (protein biosynthesis and nucleoside, nucleotide, and nucleic acid metabolism) and molecular function (ribosomal protein) were differentially regulated and stage-specific (Tables 1 and 2).

**Table 2:** Ontological classes of differentially expressed genes in long-standing rheumatoid arthritis versus controls

	Gene regulation	Long-standing rheumatoid arthritis versus controls	*P *value
Biological processes			
	Up (cluster 2)		
		Pheromone response	0.002
		Chemosensory perception	0.002
		Pyrimidine metabolism	0.03
	Down (cluster 1)		
		Fatty acid beta oxidation	0.003
		Protein biosynthesis^a^	0.007
		Nucleoside, nucleotide, and nucleic acid metabolism^a^	0.009
		Constitutive exocytosis	0.02
		Vitamin biosynthesis	0.03
		Amino acid transport	0.04
		Fatty acid metabolism	0.04
Molecular functions			
	Up (cluster 2)		
		Serine protease	0.003
		Interleukin receptor	0.02
		Protease	0.03
		Nucleotide kinase	0.04
		Cytokine receptor	0.04
	Down (cluster 1)		
		Storage protein	0.002
		Ribosomal protein^a^	0.01
		Metalloprotease inhibitor^a^	0.02
		Amino acid transporter	0.02
		Extracellular matrix linker protein	0.02
		Other receptor	0.04
Pathways			
	Up (cluster 2)		
		Salvage pyrimidine deoxyribonucleotides^a^	0.003
		Vasopressin synthesis	0.005
		p38 MAPK pathway	0.03
	Down (cluster 1)		
		Vitamin D metabolism and pathway	0.02
		Histamine receptor-mediated signaling pathway	0.04
		Enkephalin release	0.04

The gene-ontology terms used are those of the PANTHER (Protein ANalysis THrough Evolutionary Relationships) database. ^a^Common ontological classes found in both comparisons: early rheumatoid arthritis (RA) or long-standing RA versus controls (see also Table 1). MAPK, mitogen-activated protein kinase.

## Discussion

A previous report [2] already showed that RA is a heterogeneous disease with distinct molecular forms, but to date, few studies have focused on different RA stages [17]. Herein, we showed, for the first time, that gene-expression profiling in synovia from patients with early untreated RA and those with treated LS RA revealed opposite gene regulations, suggesting the involvement of different pathophysiological mechanisms during the disease course. Moreover, the novel comparison of the gene-expression levels in early or LS RA with those observed in healthy (mechanical or traumatic lesions) synovia brings information that is more appropriate than that obtained with osteoarthritis synovia (used as the reference in most of the published studies). The synovial biopsies, taken from sites of active RA-associated synovitis, enabled us to examine RA molecular processes. In LS RA, because the biopsied joints still harbored active disease, it seems likely that the pathophysiology and molecular processes were not influenced by methotrexate or that, at worst, they were weakly influenced by methotrexate.

The small number of samples and the fact that we did not perform a pangenomic study limit the possibility of making any distinction between RA with and RA without anti-cyclic citrullinated peptide autoantibodies and thus of drawing putative pathophysiological conclusions. Indeed, the 12,000 cDNA probes covering our array were selected on the basis of tissue-preferred expression in liver and corresponded to genes with liver-restricted expression (10% of the probes) and genes with combined hepatic and broad expression (the other 90%). Moreover, preliminary comparison with a pangenomic array indicated that our array is able to detect more than two thirds of the genes from healthy synovia (data not shown). Because this restrictive procedure cannot measure every transcript expressed in the tissue, it is not intended to provide a genome-wide view of the RA-associated gene dysregulations. Nevertheless, our approach seems quite acceptable, as our major task here was a preliminary study of molecular profiles in early and LS RA versus control synovia.

These limitations aside, our observations identified several differences in ontological processes, thereby also suggesting several putative differences in the RA pathophysiological mechanisms as a function of disease progression. In addition, the distinct biological processes identified here according to RA stage are compatible with our knowledge of its pathophysiology. Indeed, the genes linked to the biological processes, referred to as MHC class II-mediated immunity, immunity and defense, and T cell-mediated immunity, which represent the hallmarks of immune system activation, were upregulated in early RA. The antibacterial response, T cell-mediated immunity, and macrophage-mediated immunity are also the main functional and biological processes involved at RA onset. Conversely, genes involved in the cell cycle, apoptosis inhibition, and granulocyte-mediated immunity were upregulated in LS RA, suggesting the involvement of a proliferative process. By identifying dysregulated genes in early and LS RA as compared with healthy synovia, we provide arguments that the biological processes, molecular functions, and pathways differed according to the stage.

However, the results of the comparisons, early or LS RA versus controls, seem to be contradictory for some biological processes (Tables 1 and 2) (Table S3 in Additional data file 3). For example, the genes involved in T cell-mediated immunity were upregulated in early versus LS RA (*HLAB*, *HLADRB1*, *CLEC4M*, and so on) but were downregulated in early versus controls (*IFFG1*, *LTB*, and so on). These findings are not discordant, because the genes belonging to each class were different and did not overlap. Moreover, some processes/pathways are common to early and LS RA but the expressions of the genes belonging to these classes are subjected to opposite regulations. For instance, genes for protein biosynthesis, for nucleoside, nucleotide, and nucleic acid metabolism, and for ribosomal proteins were upregulated in early RA versus controls but were downregulated in LS RA versus controls. These opposite regulations could reflect disease processes involved during the different stages of disease. On the contrary, genes encoding metalloprotease inhibitor and salvage pyrimidine deoxyribonucleotide ontological classes were regulated similarly in early and LS RA. This overlap could reflect an RA-specific mechanism, regardless of the disease stage. But when we compared the gene sets involved in that mechanism and those dysregulated in RA versus previously published controls [2,17,18], we found only three genes in common:*CEBPβ*, *HCLS1*, and *TIMP1*.

Notably, the age differences among controls and early RA and LS RA patients might influence the results. Indeed, de Magalhães and colleagues [19] reported that gene expression relative to inflammation, immune response, lysosome, energy metabolism, cell cycle, and cellular senescence could be affected by the aging process. Consequently, it is conceivable that differences observed herein could be explained, at least in part, by age rather than RA. This limitation is very difficult to avoid in clinical practice because restricting selection of RA patients to those of the same age but with different RA stages represents a real, if not impossible, challenge.

Previously, Olsen and colleagues [20] performed a similar study on early and LS RA but they used peripheral blood mononuclear cells (PBMCs) from patients being treated with DMARDs or corticosteroids. The authors found that early RA was associated with a distinct gene-expression profile in PBMCs, with a signature that reflected an immune response to an unidentified infectious agent. Despite the differences in study designs, some families of genes (cytochrome *P*450, zinc-finger protein, chemokine receptors, MHC class II, and *RAS *oncogene family) were common to their study and ours. For example, the *S100A10 *gene was downregulated in both early RA PBMCs and synovial tissues, whereas *HLA-DPA1*, *B2M*, and *ARHGDIB *were dysregulated in early RA PBMCs and synovial tissues. These latter discrepancies might be explained by the differences between the tissues (PBMCs versus synovia) and the controls (vaccinated versus healthy donors) used in the two studies. Even though these two studies are not completely comparable, some of their findings converge, pointing to the involvement of stress responses and defense mechanisms in early RA, and are therefore complementary, contributing to a better understanding of the different RA stages.

## Conclusions

The results of our microarray analysis show that the RA molecular signature changes during disease progression. A better understanding of the pathophysiological mechanisms involved could contribute to the identification of targets that are more specific and, hence, therapeutic tools that are more appropriate. This approach might represent a novel way to improve and optimize RA therapeutic strategies.

## Abbreviations

aRNA: antisense RNA; DMARD: disease-modifying antirheumatic drug; LS: long-standing; MHC: major histocompatibility complex; PANTHER: Protein ANalysis THrough Evolutionary Relationships; PBMC: peripheral blood mononuclear cell; RA: rheumatoid arthritis.

## Competing interests

The authors declare that they have no competing interests.

## Authors' contributions

TL had full access to all of the study data and takes responsibility for the integrity of the data and the accuracy of the data analysis. He helped conceive of the study and participated in its design and coordination and helped perform or collect the synovial biopsies and clinical data, analyze and interpret biological data, draft the manuscript, and perform the statistical analyses. OV and JPS helped conceive of the study and participated in its design and coordination and helped analyze and interpret biological data and draft the manuscript. XLL helped conceive of the study and participated in its design and coordination and helped draft the manuscript. MH, XA, NB, IAA, and GC helped perform or collect the synovial biopsies and clinical data. CB and CD helped analyze and interpret biological data, draft the manuscript, and perform the statistical analyses. FT helped analyze and interpret biological data. MD helped draft the manuscript. All authors read and approved the final manuscript.

## Supplementary Material

Additional data file 1 Word file containing a table with the baseline characteristics of controls and rheumatoid arthritis (RA) patients. Click here for file

Additional data file 2 Adobe file containing a table listing the 719 genes differentially expressed at early RA versus long-standing RA. Click here for file

Additional data file 3 Word file containing a table listing the ontological classes of differentially expressed genes in early RA *versus *long-standing RA. Click here for file

Additional data file 4 Adobe file containing a table listing the 116 genes differentially expressed at early RA versus controls. Click here for file

Additional data file 5 Adobe file containing a table listing the 52 genes differentially expressed at long-standing RA versus controls. Click here for file
