# Supplementary material for: Early and long-standing rheumatoid arthritis: distinct molecular signatures identified by gene-expression profiling in synovia
Source: Arthritis Res Ther. 2009 Jun 29;11(3):R99. doi: 10.1186/ar2744 (PMC2714155; doi:10.1186/ar2744)
Supplement: Additional data file 1 — Word file containing a table with the baseline characteristics of controls and rheumatoid arthritis (RA) patients. [file ar2744-S1.doc]

**Table S1** Baseline characteristics of controls and rheumatoid arthritis (RA) patients

Subject Age Sex Disease RF Structural

duration and/or anti-CCP damage

Control

C1 22 F NA NA NA

C2 26 M NA NA NA

C3 25 M NA NA NA

C4 33 F NA NA NA

C5 Missing M NA NA NA

C6 27 M NA NA NA

C7 29 M NA NA NA

Early-RA patient

eRA1 32 F Missing – +

eRA2 26 F 5 months – –

eRA3 29 M 9 months – +

eRA4 40 M 6 months + –

Long-standing RA patient

LS RA1 42 F 10 years – +

LS RA2 45 F 21 years + +

LS RA3 42 F 4 years + +

LS RA4 64 F 11 years + +

RF: rheumatoid factors; anti-CCP: anti-cyclic citrullinated peptide autoantibodies; NA: not applicable; F: female; M: male; +: present; –: absent.
