# Supplementary material for: Early and long-standing rheumatoid arthritis: distinct molecular signatures identified by gene-expression profiling in synovia
Source: Arthritis Res Ther. 2009 Jun 29;11(3):R99. doi: 10.1186/ar2744 (PMC2714155; doi:10.1186/ar2744)
Supplement: Additional data file 2 — Adobe file containing a table listing the 719 genes differentially expressed at early RA versus long-standing RA. [file ar2744-S2.pdf]

Table S2 List of 719 genes differentially expressed at early RA (eRA) *versus* long-standing RA (LSRA)

uniqID	IMAGE Id	UGCluster	Name	Symbol	eRA Group mean	LS RA Group mean	Fold change	P value
13	114385	Hs.190621	Data not found		-0.777	-0.289	2.69	<1E-04
24	123172	Data not found	Data not found		-0.101	-0.566	0.18	<1E-04
31	114702	Hs.456557	Chromosome 1 open reading frame 164	C1orf164	0.425	-0.341	-1.25	<1E-04
32	120866	Hs.269577	Data not found		0.342	-0.556	-0.62	<1E-04
41	120075	Hs.17483	CD4 molecule	CD4	0.435	-0.568	-0.77	<1E-04
42	122764	Hs.33642	Archain 1	ARCN1	0.033	-0.721	-0.05	<1E-04
50	120918	Hs.523443	Hemoglobin, beta	HBB	0.381	-0.631	-0.6	<1E-04
61	127380	Hs.20205	Hemoglobin, beta pseudogene 1	HBBP1	0.622	-0.105	-5.93	<1E-04
106	128226	Hs.159525	Ketohexokinase (fructokinase)	KHK	0.296	-0.170	-1.75	<1E-04
165	110538	Hs.530943	Data not found		-0.461	0.026	-17.41	<1E-04
170	193197	Data not found	Data not found		0.261	-0.033	-7.97	<1E-04
193	114910	Data not found	Data not found		-0.556	-0.138	4.03	<1E-04
221	122016	Hs.509872	Data not found		0.911	-0.226	-4.03	<1E-04
239	122500	In multiple clusters	In multiple clusters		0.048	-0.200	-0.24	<1E-04
240	126241	In multiple clusters	Serpin peptidase inhibitor, clade E, member 1	SERPINE1	0.182	-0.140	-1.3	<1E-04
241	151951	Hs.513491	Yippee-like 3 (Drosophila)	YPEL3	0.215	0.029	7.5	<1E-04
242	195723	Hs.77741	In multiple clusters		0.371	0.027	13.48	<1E-04
247	115082	Hs.16247	Data not found		0.229	-0.282	-0.81	<1E-04
249	124759	Hs.564321	Transcribed locus		0.436	-0.388	-1.13	<1E-04
251	194959	Hs.473554	Data not found		0.806	-0.044	-18.19	<1E-04
256	116644	Hs.50308	Huntingtin interacting protein 2	HIP2	1.390	-0.119	-11.72	<1E-04
257	122020	Hs.443057	CD53 molecule	CD53	0.028	-0.362	-0.08	<1E-04
258	126699	Hs.19872	Homo sapiens, clone IMAGE:3883659, mRNA		0.078	-0.413	-0.19	<1E-04
265	115642	Data not found	Data not found		0.362	-0.292	-1.24	<1E-04
275	122022	Data not found	Data not found		0.186	-0.176	-1.06	<1E-04
298	82881	Hs.525264	Complement component 1, r subcomponent-like	C1RL	0.353	0.058	6.14	<1E-04
373	114714	Hs.534255	Beta-2-microglobulin	B2M	0.425	-0.133	-3.19	<1E-04
395	195766	Hs.479693	Transcribed locus		0.473	0.040	11.74	<1E-04

409	115772	Data not found	Data not found		0.456	-0.036	-12.66	<1E-04
419	122435	Hs.434253	HCG2003663		0.594	-0.083	-7.12	<1E-04
446	121297	Hs.500375	Ectonucleoside triphosphate diphosphohydrolase 6	ENTPD6	1.083	0.073	14.88	<1E-04
502	127831	Hs.350268	Interferon regulatory factor 2 binding protein 2	IRF2BP2	-0.393	0.076	-5.14	<1E-04
508	113984	Hs.12102	Sorting nexin 3	SNX3	0.393	-0.069	-5.66	<1E-04
595	61387	Hs.558493	Taste receptor, type 2, member 14	TAS2R14	0.198	-0.456	-0.43	<1E-04
604	77923	Hs.149035	Transcribed locus		0.316	-0.043	-7.43	<1E-04
623	109097	Hs.397609	Ribosomal protein S16	RPS16	-0.146	-0.722	0.2	<1E-04
636	123215	In multiple clusters	Full length insert cDNA clone YR55D08		0.531	0.144	3.69	<1E-04
637	127607	Hs.79018	Data not found		0.322	-0.044	-7.38	<1E-04
679	114748	Hs.175120	Hypothetical protein FLJ11286	FLJ11286	0.190	-0.112	-1.69	<1E-04
683	195530	Hs.534330	Metallothionein 1E	MT1E	0.418	-0.064	-6.5	<1E-04
714	112471	Hs.180909	Peroxiredoxin 1	PRDX1	0.419	-0.612	-0.68	<1E-04
793	84418	Hs.238996	In multiple clusters		0.493	0.079	6.27	<1E-04
811	83707	Hs.435515	Hypothetical protein LOC255167	LOC255167	0.710	0.261	2.72	<1E-04
812	110227	Hs.567389	Mitogen-activated protein kinase 1	MAPK1	0.041	-0.228	-0.18	<1E-04
840	112077	Hs.412370	Ribosomal protein L9	RPL9	-0.141	-0.746	0.19	<1E-04
849	113630	Data not found	Data not found		0.355	-0.218	-1.62	<1E-04
859	115223	Hs.280604	Data not found		0.448	0.125	3.59	<1E-04
870	123612	Hs.368944	Data not found		-0.390	-0.019	20.35	<1E-04
899	194314	Hs.249181	Data not found		0.536	0.010	51.43	<1E-04
904	114076	Hs.117067	Data not found		0.268	0.028	9.64	<1E-04
1021	114926	Data not found	Data not found		0.965	-0.361	-2.67	<1E-04
1029	110608	In multiple clusters	Homogentisate 1,2-dioxygenase	HGD	0.337	0.033	10.08	<1E-04
1099	84101	Hs.318894	G protein-coupled receptor 126	GPR126	0.460	0.070	6.6	<1E-04
1106	196202	Hs.538189	Transcribed locus		-0.857	-0.077	11.15	<1E-04
1112	121347	In multiple clusters	In multiple clusters		0.524	-0.509	-1.03	<1E-04
1126	81796	Hs.1360	Cytochrome P450, family 2, subfamily B, polypeptide 6	CYP2B6	0.386	-0.174	-2.22	<1E-04
1148	121656	Hs.432690	Solute carrier family 39 (zinc transporter), member 9	SLC39A9	0.378	-0.007	-55.61	<1E-04
1155	113265	Hs.355708	Transmembrane protein 127	TMEM127	0.479	-0.004	-122.36	<1E-04

1156	115880	Hs.559340	Hypothetical protein LOC285074	LOC285074	-0.099	-0.433	0.23	<1E-04
1157	122872	Hs.120817	Data not found		0.356	-0.102	-3.5	<1E-04
1163	109718	Data not found	Data not found		0.478	0.001	343.93	<1E-04
1167	124828	Hs.161000	Data not found		2.065	0.215	9.62	<1E-04
1174	116322	Data not found	Data not found		0.441	0.018	24.02	<1E-04
1187	195586	Hs.476982	Data not found		0.218	-0.129	-1.69	<1E-04
1191	113316	Hs.472838	In multiple clusters		0.171	-0.419	-0.41	<1E-04
1208	110218	Hs.283884	Clone IMAGE:110218 mRNA sequence		0.189	-0.072	-2.62	<1E-04
1223	195472	Hs.406184	FGFR1 oncogene partner 2	FGFR1OP2	0.632	0.046	13.82	<1E-04
1232	196627	Hs.138499	Transcribed locus		1.160	0.047	24.69	<1E-04
1244	110635	In multiple clusters	In multiple clusters		0.305	-0.610	-0.5	<1E-04
1253	109050	Hs.446427	In multiple clusters		0.580	-0.605	-0.96	<1E-04
1259	195381	Hs.549777	Full length insert cDNA clone YP97D11		0.559	0.104	5.35	<1E-04
1290	112126	In multiple clusters	Tumor protein, translationally-controlled 1	TPT1	0.034	-0.985	-0.03	<1E-04
1310	121393	Hs.17892	Transcribed locus		0.242	-0.224	-1.08	<1E-04
1352	108246	Hs.431850	Data not found		0.493	-0.031	-15.76	<1E-04
1418	120995	Hs.522932	Nuclear receptor coactivator 4	NCOA4	0.097	-0.650	-0.15	<1E-04
1488	111654	Hs.75969	Proline-rich nuclear receptor coactivator 1	PNRC1	-0.060	-0.354	0.17	<1E-04
1517	122909	Data not found	Data not found		1.112	-0.092	-12.02	<1E-04
1562	121731	Hs.131459	Cytochrome P450, family 4, subfamily F, polypeptide 12	CYP4F12	0.077	-0.199	-0.39	<1E-04
1578	112442	Hs.509447	Data not found		0.495	-0.016	-30.45	<1E-04
1589	122540	Hs.497967	Data not found		-0.200	-0.349	0.57	<1E-04
1595	109766	Hs.469331	START domain containing 7	STARD7	0.245	-0.497	-0.49	<1E-04
1630	83045	Data not found	Data not found		0.611	0.031	19.67	<1E-04
1642	116618	Hs.413208	AarF domain containing kinase 1	ADCK1	0.674	-0.103	-6.51	<1E-04
1643	122174	Hs.389171	PTEN induced putative kinase 1	PINK1	0.032	-0.144	-0.22	<1E-04
1660	116732	Data not found	Data not found		1.537	0.470	3.27	<1E-04
1663	37354	Hs.188757	Potassium channel tetramerisation domain containing 20	KCTD20	0.378	0.123	3.07	<1E-04
1664	196645	Hs.268849	Glyoxalase I	GLO1	1.164	0.133	8.75	<1E-04
1673	194753	Hs.493272	Ankyrin repeat domain 15	ANKRD15	1.084	0.113	9.6	<1E-04

1677	113347	Hs.111903	Data not found		0.489	-0.007	-69.95	<1E-04
1678	116431	Data not found	Data not found		0.342	-0.566	-0.6	<1E-04
1715	122131	Hs.246112	Activating signal cointegrator 1 complex subunit 3-like 1	ASCC3L1	0.362	0.116	3.13	<1E-04
1720	82173	Hs.15400	Dexamethasone-induced transcript	DEXI	1.882	1.064	1.77	<1E-04
1756	78051	Hs.154078	Lipopolysaccharide binding protein	LBP	0.547	-0.006	-93.72	<1E-04
1787	120320	Hs.532399	Zinc finger CCCH-type containing 11A	ZC3H11A	0.078	-0.243	-0.32	<1E-04
1817	194401	Hs.381058	Data not found		0.682	0.150	4.53	<1E-04
1844	191725	Data not found	Data not found		0.649	0.048	13.43	<1E-04
1893	111388	Hs.499038	Tyrosine 3-monooxygenase/tryptophan 5-monooxygenase	YWHAZ	0.391	-0.259	-1.51	<1E-04
1907	194398	Hs.435136	Thioredoxin	TXN	0.406	-0.622	-0.65	<1E-04
1961	195533	Hs.538417	Transcribed locus		1.403	0.092	15.2	<1E-04
1983	113381	Hs.500159	Similar to Estradiol 17-beta-dehydrogenase 1	LOC643646	0.392	-0.048	-8.23	<1E-04
1985	122161	Hs.124147	Data not found		0.324	0.073	4.46	<1E-04
1995	125271	Hs.529892	Sequestosome 1	SQSTM1	-0.647	-0.329	1.97	<1E-04
2021	122698	Hs.5148	Data not found		0.289	-0.153	-1.88	<1E-04
2044	83075	Hs.369920	RAP1B, member of RAS oncogene family	RAP1B	0.293	-0.398	-0.74	<1E-04
2096	197109	Hs.99886	Transcribed locus		0.529	0.104	5.07	<1E-04
2131	129909	In multiple clusters	In multiple clusters		-0.291	0.193	-1.51	<1E-04
2147	122590	In multiple clusters	In multiple clusters		0.213	-0.063	-3.38	<1E-04
2173	114625	Hs.530727	CDNA FLJ32454 fis, clone SKNMC1000007		0.569	-0.426	-1.34	<1E-04
2224	78956	Hs.567289	Data not found		0.494	-0.004	-136.2	<1E-04
2231	194049	Hs.380277	Data not found		0.467	0.050	9.41	<1E-04
2244	111843	Data not found	Data not found		0.247	-0.118	-2.1	<1E-04
2249	194464	Hs.370834	Transcribed locus		0.344	-0.043	-7.95	<1E-04
2255	120372	Data not found	Data not found		0.225	-0.214	-1.05	<1E-04
2308	114208	Hs.546239	Alpha-2-glycoprotein 1, zinc-binding	AZGP1	0.413	-0.522	-0.79	<1E-04
2363	120717	Hs.336994	Data not found		0.376	0.107	3.53	<1E-04
2371	115004	Data not found	Data not found		0.209	-0.100	-2.08	<1E-04
2379	113573	Hs.37706	Data not found		0.086	-0.761	-0.11	<1E-04
2407	115322	Hs.191887	Sec61 beta subunit	SEC61B	-0.140	-0.498	0.28	<1E-04

2413	83860	Data not found	Data not found		0.190	-0.175	-1.09	<1E-04
2438	196350	Hs.482103	Data not found		0.478	-0.041	-11.54	<1E-04
2474	196379	Hs.391860	Data not found		0.170	-0.223	-0.76	<1E-04
2479	115285	Hs.252828	Processing of precursor 1, ribonuclease P/MRP subunit	POP1	0.110	-0.230	-0.48	<1E-04
2512	81927	Hs.474436	Hermansky-Pudlak syndrome 4	HPS4	0.636	0.133	4.77	<1E-04
2540	110392	Hs.247077	Ras homolog gene family, member A	RHOA	0.544	-0.719	-0.76	<1E-04
2618	193322	Hs.520048	Major histocompatibility complex, class II, DR alpha	HLA-DRA	0.234	-0.545	-0.43	<1E-04
2650	114259	Hs.186544	Data not found		0.287	-0.083	-3.45	<1E-04
2668	114248	Hs.7720	Dynein, cytoplasmic 1, heavy chain 1	DYNC1H1	0.384	-0.692	-0.56	<1E-04
2756	108252	In multiple clusters	Prosaposin	PSAP	0.479	-1.305	-0.37	<1E-04
2777	119636	Hs.444915	Solute carrier family 1, member 1	SLC1A1	0.813	0.241	3.37	<1E-04
2872	82301	Hs.515835	Sulfotransferase family, cytosolic, 2A, member 1	SULT2A1	0.592	0.000	-1899.59	<1E-04
2876	121976	Hs.25647	V-fos FBJ murine osteosarcoma viral oncogene	FOS	-0.084	-0.496	0.17	<1E-04
2924	196428	Hs.486095	CDNA FLJ11366 fis, clone HEMBA1000282		1.581	0.255	6.21	<1E-04
2957	122845	Hs.502765	Hypothetical LOC643517	LOC643517	-0.255	0.175	-1.46	<1E-04
2962	83074	Data not found	Transcribed locus		0.492	-0.038	-13.02	<1E-04
2963	110059	Hs.507971	Data not found		-0.056	-0.745	0.08	<1E-04
2969	195547	Hs.136829	Data not found		0.504	0.005	95.57	<1E-04
3004	128852	Hs.512001	Glycosylphosphatidylinositol specific phospholipase D1	GPLD1	-0.283	0.280	-1.01	<1E-04
3013	42197	Hs.79101	Cyclin G1	CCNG1	0.248	-0.497	-0.5	<1E-04
3022	129239	In multiple clusters	In multiple clusters		0.083	-0.938	-0.09	<1E-04
3029	120569	Data not found	Data not found		0.394	0.018	22.45	<1E-04
3074	121209	Hs.191330	CDNA FLJ42255 fis, clone TKIDN2009889		0.214	-0.207	-1.03	<1E-04
3082	114270	Hs.431099	PDZK1 interacting protein 1	PDZK1IP1	0.186	-0.106	-1.75	<1E-04
3092	121591	Hs.439815	Hepatitis B virus x interacting protein	HBXIP	0.297	-0.373	-0.8	<1E-04
3095	194560	Hs.23751	ADAM metallopeptidase with thrombospondin	ADAMTS19	0.374	-0.233	-1.61	<1E-04
3116	108293	Hs.150580	Eukaryotic translation initiation factor 1	EIF1	0.046	-0.971	-0.05	<1E-04
3164	121596	Data not found	Data not found		0.492	-0.006	-86.74	<1E-04
3170	108405	Hs.439552	Eukaryotic translation elongation factor 1 alpha 1	EEF1A1	0.027	-1.044	-0.03	<1E-04
3179	108930	Hs.522767	Solute carrier family 25, member 5	SLC25A5	-0.111	-0.948	0.12	<1E-04

3190	113922	Hs.567377	Data not found		0.049	-0.483	-0.1	<1E-04
3214	78594	Hs.172028	In multiple clusters		0.192	-0.172	-1.12	<1E-04
3227	120612	Data not found	Data not found		0.751	0.178	4.21	<1E-04
3246	126672	Hs.9754	Activating transcription factor 5	ATF5	0.383	-0.079	-4.84	<1E-04
3257	194856	Hs.499000	Data not found		1.147	0.035	32.92	<1E-04
3298	116914	Hs.495511	MRNA; cDNA DKFZp686O02112		-0.056	-1.231	0.05	<1E-04
3299	122307	Hs.555925	Armadillo repeat containing 8	ARMC8	0.281	-0.006	-45.66	<1E-04
3302	196827	Hs.546286	Ribosomal protein S3	RPS3	0.599	-0.115	-5.21	<1E-04
3311	195299	Hs.504877	Rho GDP dissociation inhibitor (GDI) beta	ARHGDIB	0.296	-0.202	-1.46	<1E-04
3318	126952	Hs.443977	Data not found		0.221	-0.118	-1.87	<1E-04
3319	161128	Hs.522568	Phosphatidylinositol-specific phospholipase CXD1	PLCXD1	0.471	-0.015	-31.52	<1E-04
3336	126565	In multiple clusters	Protein disulfide isomerase family A, member 6	PDIA6	0.240	-0.234	-1.03	<1E-04
3345	125304	Hs.75599	Serpin peptidase inhibitor, clade C, member 1	SERPINC1	0.353	-0.160	-2.21	<1E-04
3346	130011	In multiple clusters	Data not found		0.385	-0.153	-2.51	<1E-04
3349	84348	Data not found	Data not found		0.551	0.080	6.88	<1E-04
3413	109701	Data not found	Data not found		0.452	0.097	4.65	<1E-04
3415	115434	Hs.10739	Transcribed locus		0.684	-0.196	-3.48	<1E-04
3422	110420	In multiple clusters	In multiple clusters		-0.031	-0.839	0.04	<1E-04
3424	116816	Hs.541269	Data not found		0.255	-0.210	-1.21	<1E-04
3462	346009	In multiple clusters	Phosphofructokinase, liver	PFKL	-1.926	-0.592	3.25	<1E-04
3469	244659	Hs.133352	Data not found		0.134	-0.136	-0.98	<1E-04
3567	211116	Hs.420272	H2A histone family, member Y	H2AFY	0.675	-0.128	-5.27	<1E-04
3583	198826	Hs.533787	KIAA0286 protein	KIAA0286	3.193	1.926	1.66	<1E-04
3601	198828	Hs.520814	Tensin 3	TNS3	1.816	0.330	5.5	<1E-04
3603	212252	Hs.536876	CDC42 small effector 2	CDC42SE2	1.075	0.501	2.15	<1E-04
3619	198542	Hs.425125	Ribosomal protein L29	RPL29	0.528	0.038	13.79	<1E-04
3650	292534	Hs.510375	Nucleoporin 43kDa	NUP43	-0.364	0.287	-1.27	<1E-04
3703	245332	Hs.534293	Serpin peptidase inhibitor, clade A, member 3	SERPINA3	-0.392	0.061	-6.45	<1E-04
3721	245736	Hs.143347	Data not found		-0.578	0.106	-5.43	<1E-04
3739	244875	Hs.533013	Cystathionine-beta-synthase	CBS	-0.512	-0.045	11.49	<1E-04

3757	245289	Hs.403790	Solute carrier family 25, member 28	SLC25A28	-0.273	0.065	-4.19	<1E-04
3770	125073	Hs.397609	Ribosomal protein S16	RPS16	0.106	-0.740	-0.14	<1E-04
3779	120874	Hs.368078	DnaJ (Hsp40) homolog, subfamily A, member 2	DNAJA2	-0.121	-0.880	0.14	<1E-04
3791	206895	Hs.292754	Transcribed locus		0.865	-0.015	-57.12	<1E-04
3817	202769	Data not found	Data not found		-0.028	0.426	-0.07	<1E-04
3819	234137	Hs.559043	Hypothetical gene supported by AF086559; BC065734	LOC441383	0.490	0.269	1.82	<1E-04
3847	245750	Hs.512155	Haptoglobin-related protein	HPR	-0.431	0.094	-4.58	<1E-04
3856	254090	Hs.496710	Stromal antigen 2	STAG2	-0.398	0.316	-1.26	<1E-04
3935	206115	Hs.232021	Transcribed locus		-0.647	-0.139	4.66	<1E-04
4001	279847	Hs.6790	DnaJ (Hsp40) homolog, subfamily B, member 9	DNAJB9	-0.208	0.343	-0.61	<1E-04
4007	206164	Data not found	Data not found		0.143	-0.546	-0.26	<1E-04
4015	198503	Hs.474584	Aldo-keto reductase family 1, member A1	AKR1A1	0.223	-0.078	-2.86	<1E-04
4037	275985	Hs.529438	Hypermethylated in cancer 2	HIC2	-0.451	0.185	-2.44	<1E-04
4045	243350	Hs.268788	In multiple clusters		-0.553	0.223	-2.48	<1E-04
4291	21771	Hs.504828	DEAD (Asp-Glu-Ala-Asp) box polypeptide 47	DDX47	1.069	0.019	56.75	<1E-04
4296	233823	Hs.484371	Mitogen-activated protein kinase 9	MAPK9	-0.528	0.072	-7.36	<1E-04
4306	258448	Hs.536567	Hypothetical protein LOC728730	LOC728730	1.098	0.139	7.91	<1E-04
4316	294118	Data not found	Data not found		-0.535	-0.009	60.7	<1E-04
4324	241937	Hs.446118	Data not found		0.188	-0.189	-1	<1E-04
4333	243049	Data not found	Data not found		0.482	0.054	8.91	<1E-04
4335	364825	Hs.523913	CDNA FLJ30156 fis, clone BRACE2000487		0.375	0.076	4.93	<1E-04
4369	244306	Hs.22500	Data not found		-0.556	-0.033	17.02	<1E-04
4442	293654	Hs.370689	Data not found		-0.192	0.032	-6.04	<1E-04
4490	109437	Hs.14894	Trans-golgi network protein 2	TGOLN2	-0.773	-0.156	4.96	<1E-04
4541	298615	Hs.79387	Proteasome (prosome, macropain)	PSMC5	0.146	-0.419	-0.35	<1E-04
4546	200881	Hs.415576	KIAA1450 protein	KIAA1450	0.022	-0.288	-0.08	<1E-04
4550	294507	Hs.529925	Ubiquitin protein ligase E3 component n-recognin 2	UBR2	0.506	-0.194	-2.61	<1E-04
4552	430061	Hs.34576	Data not found		0.065	-0.342	-0.19	<1E-04
4568	294171	Hs.292493	X-ray repair complementing defective repair	XRCC6	-0.420	-0.107	3.93	<1E-04
4575	240050	Data not found	Data not found		0.418	0.033	12.59	<1E-04

4598	125723	Hs.514107	Chemokine (C-C motif) ligand 3	CCL3	1.530	0.786	1.95	<1E-04
4636	201203	Hs.464416	Ubiquitin specific peptidase 14	USP14	0.393	0.132	2.99	<1E-04
4646	210812	Hs.82028	Data not found		0.340	0.037	9.12	<1E-04
4708	200899	Hs.558319	Aldo-keto reductase family 1, member C2	AKR1C2	-0.464	0.109	-4.24	<1E-04
4710	230419	Hs.167614	Data not found		-0.026	0.286	-0.09	<1E-04
4759	418248	Hs.567512	Transcribed locus		0.111	-0.130	-0.85	<1E-04
4772	203549	Hs.553105	Data not found		0.421	0.140	3.02	<1E-04
4852	199716	Hs.271643	Data not found		0.190	-0.031	-6.18	<1E-04
4928	294138	Data not found	Data not found		-0.549	0.081	-6.8	<1E-04
4978	200880	Hs.558453	ATP synthase, H+ transporting, mitochondrial F0	ATP5L	0.169	0.027	6.33	<1E-04
5026	252358	Hs.159971	Der1-like domain family, member 3	DERL3	1.674	1.318	1.27	<1E-04
5052	230432	Data not found	Data not found		-0.046	0.364	-0.13	<1E-04
5120	126438	Data not found	Data not found		0.534	0.097	5.51	<1E-04
5140	200826	Hs.174021	Data not found		0.019	0.266	0.07	<1E-04
5147	120643	Hs.382306	Data not found		0.511	0.117	4.37	<1E-04
5208	345722	Hs.491308	RAB25, member RAS oncogene family	RAB25	-0.368	-0.027	13.41	<1E-04
5213	206785	Hs.544913	Data not found		2.285	1.279	1.79	<1E-04
5234	292683	Hs.543062	Transcribed locus		0.476	0.016	29.89	<1E-04
5251	243956	Data not found	Data not found		-0.268	0.493	-0.54	<1E-04
5275	199015	Data not found	Data not found		1.651	0.101	16.41	<1E-04
5336	22328	Hs.512914	NMDA receptor regulated 1-like	NARG1L	0.775	0.275	2.81	<1E-04
5347	197872	Data not found	Data not found		-0.348	0.321	-1.09	<1E-04
5360	292697	Hs.370147	Centrosome and spindle pole associated protein 1	CSPP1	-0.870	-0.062	14.07	<1E-04
5381	120306	Hs.135087	In multiple clusters		0.418	0.113	3.71	<1E-04
5411	207049	Hs.496542	Ring finger protein 128	RNF128	-0.575	-0.018	31.55	<1E-04
5439	239596	Hs.489105	Data not found		1.873	1.128	1.66	<1E-04
5500	200938	Hs.158209	5'-nucleotidase, cytosolic II	NT5C2	0.505	0.164	3.08	<1E-04
5503	247165	Data not found	Data not found		0.431	0.043	10.05	<1E-04
5531	296199	Hs.423163	Transcribed locus		-0.348	0.129	-2.7	<1E-04
5556	233564	Hs.122523	Data not found		1.898	0.496	3.83	<1E-04

5594	295648	Hs.560983	Transcribed locus		-0.612	0.139	-4.4	<1E-04
5610	233806	Hs.440525	Translocase of inner mitochondrial membrane 9 homolog	TIMM9	-0.396	-0.054	7.33	<1E-04
5618	204722	Hs.24379	Trafficking protein particle complex 1	TRAPPC1	0.264	-0.418	-0.63	<1E-04
5626	200604	Hs.268787	Transcribed locus		0.531	0.099	5.37	<1E-04
5711	275793	Hs.58690	Data not found		-0.414	0.108	-3.84	<1E-04
5736	229405	In multiple clusters	Transcribed locus		-0.074	0.639	-0.12	<1E-04
5754	214795	Hs.118161	Data not found		1.637	1.279	1.28	<1E-04
5761	198780	Hs.368226	Data not found		1.530	0.134	11.4	<1E-04
5806	200204	Hs.200770	Data not found		-0.341	0.087	-3.92	<1E-04
5810	292748	Hs.488051	CSRP2 binding protein	CSRP2BP	-0.612	0.238	-2.57	<1E-04
5828	292391	Hs.536275	Transcribed locus		-0.464	0.159	-2.92	<1E-04
5863	245787	Hs.187376	Intraflagellar transport 88 homolog (Chlamydomonas)	IFT88	-0.655	-0.003	252.71	<1E-04
5878	201365	Data not found	Data not found		0.339	0.068	5	<1E-04
5887	202531	Hs.212088	Epoxide hydrolase 2, cytoplasmic	EPHX2	0.651	-0.002	-283.42	<1E-04
5901	416001	Hs.163042	Transcribed locus		-0.236	0.161	-1.47	<1E-04
5955	415621	Hs.71465	In multiple clusters		-0.594	0.118	-5.02	<1E-04
5961	239922	Hs.112877	Transcribed locus		-0.429	0.219	-1.96	<1E-04
5964	416727	Hs.58871	Transcribed locus		-0.507	0.165	-3.06	<1E-04
5989	245125	Hs.458609	Transcribed locus		-0.177	0.067	-2.64	<1E-04
6009	416440	Data not found	Data not found		-0.796	-0.064	12.35	<1E-04
6031	203389	Hs.102408	Homo sapiens, clone IMAGE:5207242, mRNA		-0.213	0.142	-1.5	<1E-04
6044	296205	Hs.118161	Chromosome 10 open reading frame 11	C10orf11	-0.697	0.256	-2.72	<1E-04
6056	110425	Hs.472038	UTP11-like, U3 small nucleolar ribonucleoprotein, (yeast)	UTP11L	-0.109	-0.704	0.15	<1E-04
6123	213679	Hs.443875	DCP2 decapping enzyme homolog (S. cerevisiae)	DCP2	1.359	0.139	9.8	<1E-04
6229	199537	In multiple clusters	In multiple clusters		-0.770	-0.275	2.8	<1E-04
6257	205647	Hs.176503	TBC1 domain family, member 1	TBC1D1	-0.270	0.057	-4.73	<1E-04
6265	202704	Hs.109655	CDNA FLJ37623 fis, clone BRCOC2014013		0.640	0.109	5.86	<1E-04
6341	297416	Hs.193792	Transcribed locus		-0.260	0.345	-0.75	<1E-04
6382	201611	Hs.488293	Data not found		-0.716	-0.137	5.21	<1E-04
6391	203390	Data not found	Data not found		1.180	0.264	4.47	<1E-04

6397	22034	Hs.469331	START domain containing 7	STARD7	0.307	-0.256	-1.2	<1E-04
6398	127982	Hs.491582	Plasminogen activator, tissue	PLAT	1.335	-0.395	-3.38	<1E-04
6409	202168	Hs.445981	Data not found		-0.228	0.252	-0.9	<1E-04
6464	210952	Hs.504517	Data not found		0.227	-0.152	-1.49	<1E-04
6515	113522	Hs.484551	Carboxypeptidase M	CPM	0.747	0.002	417.02	<1E-04
6540	345439	Data not found	Deoxyribonuclease II, lysosomal	DNASE2	0.534	0.100	5.32	<1E-04
6546	229945	Hs.558442	Programmed cell death 6 interacting protein	PDCD6IP	0.059	-0.212	-0.28	<1E-04
6564	229924	Hs.519583	Transcribed locus		0.520	0.046	11.35	<1E-04
6569	113529	Hs.189073	In multiple clusters		0.407	-0.057	-7.08	<1E-04
6583	243322	Hs.437966	Transcribed locus		-0.808	-0.233	3.47	<1E-04
6598	200626	Hs.398636	Hemoglobin, alpha 2	HBA2	-0.470	0.104	-4.53	<1E-04
6626	205127	Hs.480364	Data not found		0.522	0.130	4	<1E-04
6654	229508	Hs.505824	Sorting and assembly machinery component 50 homolog	SAMM50	0.903	0.019	47.89	<1E-04
6747	416066	Hs.64016	Data not found		0.886	-0.701	-1.26	<1E-04
6751	202233	Data not found	Data not found		0.190	-0.122	-1.56	<1E-04
6756	416862	Hs.202521	Data not found		0.538	-0.385	-1.4	<1E-04
6762	234072	Data not found	Data not found		0.070	-0.582	-0.12	<1E-04
6770	209507	Hs.298658	Data not found		-1.029	-0.192	5.35	<1E-04
6788	210488	Hs.439544	Data not found		-0.462	0.126	-3.65	<1E-04
6805	202236	Hs.5920	Glucosamine-2-epimerase/N-acetylmannosamine kinase	GNE	1.432	-0.375	-3.81	<1E-04
6815	207968	Data not found	Data not found		0.171	-0.135	-1.26	<1E-04
6820	430313	Hs.379414	Data not found		1.644	0.175	9.39	<1E-04
6859	203010	Hs.420024	Data not found		1.256	-0.063	-20.08	<1E-04
6861	234858	Hs.528833	Data not found		1.621	0.519	3.12	<1E-04
6862	248534	Hs.554795	Plexin C1	PLXNC1	-0.639	0.092	-6.91	<1E-04
6882	416876	Hs.509872	Data not found		-0.260	0.024	-10.79	<1E-04
6935	233738	Hs.30054	Coagulation factor V (proaccelerin, labile factor)	F5	0.490	-0.148	-3.32	<1E-04
6936	266409	Hs.401903	Cytochrome c oxidase subunit Va	COX5A	1.197	-0.073	-16.42	<1E-04
6941	136282	Data not found	Chromosome 16 open reading frame 72	C16orf72	0.271	-0.564	-0.48	<1E-04
6943	200714	Hs.421437	C-type lectin domain family 4, member M	CLEC4M	0.935	-0.032	-29.45	<1E-04

6945	274081	Hs.408790	Phosphatidylinositol glycan anchor biosynthesis, class P	PIGP	1.430	-0.343	-4.17	<1E-04
6952	197356	Hs.534322	Major histocompatibility complex, class II, DR beta 1	HLA-DRB1	0.558	-0.425	-1.31	<1E-04
6953	240031	Hs.525557	Serpin peptidase inhibitor, clade A, member 1	SERPINA1	0.115	-0.539	-0.21	<1E-04
6963	271781	Hs.442609	Mitochondrial ribosomal protein L38	MRPL38	-0.136	-0.657	0.21	<1E-04
6970	197675	Hs.516578	In multiple clusters		0.412	-0.049	-8.47	<1E-04
6972	264871	Hs.220950	Forkhead box O3	FOXO3	0.563	-0.242	-2.33	<1E-04
6977	132752	Hs.159118	Adenosylmethionine decarboxylase 1	AMD1	0.472	-0.594	-0.79	<1E-04
6979	200743	Hs.80756	Betaine-homocysteine methyltransferase	BHMT	0.623	-0.297	-2.1	<1E-04
6981	272913	In multiple clusters	In multiple clusters		0.054	-0.756	-0.07	<1E-04
7024	198874	Hs.351335	Transmembrane protein 143	TMEM143	0.947	-0.191	-4.97	<1E-04
7040	42648	Hs.89643	Transketolase (Wernicke-Korsakoff syndrome)	TKT	0.901	-0.114	-7.92	<1E-04
7058	41687	Hs.473648	Phosphoribosylglycinamide formyltransferase	GART	1.243	-0.078	-16	<1E-04
7067	132762	Hs.197644	Headcase homolog (Drosophila)	HECA	0.123	-0.431	-0.29	<1E-04
7085	132378	Hs.502315	Pyruvate dehydrogenase complex, component X	PDHX	-0.362	-0.662	0.55	<1E-04
7103	133173	Hs.144502	Phosphatidylinositol-4-phosphate 5-kinase, type II, γ	PIP5K2C	0.435	-0.377	-1.15	<1E-04
7129	38159	Hs.131776	Pyroglutamyl-peptidase I	PGPEP1	-1.326	-0.092	14.49	<1E-04
7131	49145	Hs.529357	CDNA FLJ13474 fis, clone PLACE1003593		-0.448	-0.204	2.2	<1E-04
7153	321774	Hs.75914	Transmembrane emp24 domain trafficking protein 2	TMED2	0.554	-0.728	-0.76	<1E-04
7169	259085	Hs.488478	Chromosome 7 open reading frame 42	C7orf42	0.067	-0.815	-0.08	<1E-04
7190	357031	Hs.437322	Tumor necrosis factor, alpha-induced protein 6	TNFAIP6	1.427	-0.360	-3.96	<1E-04
7201	38070	Hs.525391	Transcribed locus		0.459	-0.003	-178.63	<1E-04
7202	143137	Hs.520383	Syntaxin 7	STX7	0.390	-0.417	-0.94	<1E-04
7223	257557	In multiple clusters	Eukaryotic translation elongation factor 1 beta 2	EEF1B2	0.357	-0.280	-1.27	<1E-04
7226	357394	Hs.362728	15 kDa selenoprotein	sept-15	-0.133	-0.663	0.2	<1E-04
7238	143890	In multiple clusters	T cell receptor alpha locus	TRAα	0.553	-0.546	-1.01	<1E-04
7241	256788	In multiple clusters	In multiple clusters		0.378	-0.542	-0.7	<1E-04
7259	259121	Hs.249996	Pleiotropic regulator 1 (PRL1 homolog, Arabidopsis)	PLRG1	-0.055	-0.686	0.08	<1E-04
7269	293558	Hs.194121	RNA terminal phosphate cyclase-like 1	RCL1	1.460	-0.272	-5.36	<1E-04
7273	41475	Hs.523855	Syntaxin 12	STX12	1.233	0.546	2.26	<1E-04
7277	259097	Hs.250181	Golgi reassembly stacking protein 1, 65kDa	GORASP1	1.146	-0.430	-2.67	<1E-04

7278	297680	Hs.237536	5'-nucleotidase, cytosolic III-like	NT5C3L	2.187	-0.115	-18.95	<1E-04
7292	143147	Hs.282998	Ribosomal protein L41	RPL41	-0.091	-0.579	0.16	<1E-04
7295	259484	Hs.83286	KDEL (Lys-Asp-Glu-Leu) containing 2	KDELC2	1.081	-0.407	-2.65	<1E-04
7301	136987	Hs.440544	Chloride intracellular channel 4	CLIC4	1.320	-0.167	-7.9	<1E-04
7305	294132	Hs.35433	Data not found		0.247	-0.646	-0.38	<1E-04
7324	309454	Hs.466871	Plasminogen activator, urokinase receptor	PLAUR	-0.194	-0.640	0.3	<1E-04
7330	229343	In multiple clusters	Apolipoprotein A-I	APOA1	-0.630	-0.116	5.42	<1E-04
7339	206519	Hs.541856	Transcribed locus		-2.748	-0.479	5.73	<1E-04
7359	273794	In multiple clusters	In multiple clusters		0.122	-0.873	-0.14	<1E-04
7368	267582	Hs.468415	Ras homolog gene family, member Q	RHOQ	0.646	-0.299	-2.16	<1E-04
7377	271443	Hs.503178	Spectrin, beta, non-erythrocytic 1	SPTBN1	0.735	-0.293	-2.51	<1E-04
7387	300641	Hs.476365	Sterol carrier protein 2	SCP2	0.335	-0.818	-0.41	<1E-04
7418	130933	Hs.448646	Ribosomal protein L27a	RPL27A	0.082	-0.714	-0.12	<1E-04
7423	299481	Hs.134859	V-maf musculoaponeurotic fibrosarcoma oncogene	MAF	0.154	-0.512	-0.3	<1E-04
7449	277901	Hs.525146	Sodium leak channel, non-selective	NALCN	0.339	-0.605	-0.56	<1E-04
7475	240351	Hs.518265	CDV3 homolog (mouse)	CDV3	0.193	-0.553	-0.35	<1E-04
7495	299084	Hs.308709	Protein disulfide isomerase family A, member 3	PDIA3	0.601	-0.567	-1.06	<1E-04
7531	298340	Hs.517134	Chromosome 20 open reading frame 43	C20orf43	0.233	-0.507	-0.46	<1E-04
7544	42344	Hs.435479	Protein phosphatase 1H (PP2C domain containing)	PPM1H	3.744	2.679	1.4	<1E-04
7553	133650	Hs.409412	Poly (ADP-ribose) polymerase family, member 2	PARP2	0.671	-0.374	-1.8	<1E-04
7558	305182	Hs.77961	Major histocompatibility complex, class I, B	HLA-B	1.198	-0.947	-1.26	<1E-04
7574	248832	Data not found	ATPase, H+ transporting, lysosomal accessory protein 1	ATP6AP1	0.194	-0.710	-0.27	<1E-04
7583	261856	Hs.75117	Interleukin enhancer binding factor 2, 45kDa	ILF2	0.347	-0.648	-0.53	<1E-04
7611	293820	Hs.126221	Transmembrane protein 1	TMEM1	0.031	-0.672	-0.05	<1E-04
7618	212695	Hs.388255	Chromosome 16 open reading frame 61	C16orf61	0.567	-0.309	-1.83	<1E-04
7653	49164	Hs.109225	Vascular cell adhesion molecule 1	VCAM1	0.486	-1.275	-0.38	<1E-04
7698	46388	Hs.400095	Heat shock 22kDa protein 8	HSPB8	0.101	-0.569	-0.18	<1E-04
7719	291401	Hs.567441	Eukaryotic translation initiation factor 4B	EIF4B	0.721	-0.584	-1.23	<1E-04
7737	292197	Hs.78592	Eukaryotic translation initiation factor 2B, subunit 1 alpha, 26	EIF2B1	0.449	-0.523	-0.86	<1E-04
7784	327220	Hs.180178	LON peptidase N-terminal domain and ring finger 1	LONRF1	0.292	-0.698	-0.42	<1E-04

7809	271082	Hs.93780	Transcribed locus		0.444	0.074	5.98	<1E-04
7827	271876	Hs.489284	Actin related protein 2/3 complex, subunit 1B, 41kDa	ARPC1B	-0.116	-0.632	0.18	<1E-04
7836	266118	In multiple clusters	Ribosomal protein S3A	RPS3A	0.199	-0.989	-0.2	<1E-04
7927	299200	Hs.5062	Tetraspanin 3	TSPAN3	0.012	-0.876	-0.01	<1E-04
7976	130882	Hs.23363	Phosphoglucomutase 2	PGM2	-0.157	-0.490	0.32	<1E-04
7986	160515	Hs.547222	CDNA FLJ43454 fis, clone OCBBF2034906		1.197	-0.100	-12.02	<1E-04
8004	47803	Hs.20021	Vesicle-associated membrane protein 1 (synaptobrevin 1)	VAMP1	0.431	-0.252	-1.71	<1E-04
8025	291892	Hs.490394	Single-stranded DNA binding protein 1	SSBP1	0.198	-0.206	-0.96	<1E-04
8042	248066	Data not found	Ubiquitin-conjugating enzyme E2D 3	UBE2D3	-0.847	-0.021	41.1	<1E-04
8054	356722	Hs.469593	In multiple clusters		0.178	-0.093	-1.91	<1E-04
8056	34989	Hs.97858	Kinesin family member 1B	KIF1B	0.360	-0.103	-3.5	<1E-04
8059	205807	Hs.509909	Numb homolog (Drosophila)	NUMB	0.449	-0.378	-1.19	<1E-04
8071	320241	Hs.242458	Spastic paraplegia 21	SPG21	0.150	-0.519	-0.29	<1E-04
8072	347531	Hs.50550	Kelch repeat and BTB (POZ) domain containing 10	KBTBD10	0.229	-0.490	-0.47	<1E-04
8081	344815	Hs.278426	PDGFA associated protein 1	PDAP1	0.488	-0.392	-1.24	<1E-04
8088	296424	Hs.234642	Aquaporin 3 (Gill blood group)	AQP3	0.286	-0.018	-16.02	<1E-04
8095	210317	Hs.433888	RAB11B, member RAS oncogene family	RAB11B	1.362	0.225	6.06	<1E-04
8097	291059	Hs.505545	Solute carrier family 11, member 2	SLC11A2	0.508	-0.029	-17.71	<1E-04
8112	44699	Hs.7476	ATPase, H+ transporting, V0 subunit b	ATP6V0B	0.200	-0.499	-0.4	<1E-04
8113	212468	Hs.127897	Data not found		-0.664	-0.178	3.74	<1E-04
8114	248848	Hs.75160	Phosphofructokinase, muscle	PFKM	-0.872	-0.320	2.72	<1E-04
8117	344461	Hs.522500	KIAA0310	KIAA0310	-0.763	-0.083	9.22	<1E-04
8129	139166	Hs.235390	Zinc finger and SCAN domain containing 18	ZSCAN18	0.563	-0.348	-1.62	<1E-04
8150	247697	Hs.334518	Zinc finger protein 607	ZNF607	-0.023	-0.623	0.04	<1E-04
8159	261499	In multiple clusters	Ferritin, heavy polypeptide 1	FTH1	-0.066	-0.926	0.07	<1E-04
8160	297645	Hs.75535	Myosin, light chain 2, regulatory, cardiac, slow	MYL2	0.748	-0.242	-3.09	<1E-04
8168	248059	Hs.420257	Inter-alpha (globulin) inhibitor H1	ITIH1	0.219	-0.641	-0.34	<1E-04
8184	46405	Hs.307924	Male-specific lethal 3-like 1 (Drosophila)	MSL3L1	1.614	0.517	3.12	<1E-04
8193	191881	Hs.347270	Major histocompatibility complex, class II, DP alpha 1	HLA-DPA1	0.601	-0.657	-0.91	<1E-04
8202	45877	Hs.4302	Solute carrier family 29, member 4	SLC29A4	0.466	-0.497	-0.94	<1E-04

8216	327232	Hs.523185	Ribosomal protein L13a	RPL13A	-0.023	-0.696	0.03	<1E-04
8234	323977	Hs.375129	In multiple clusters		1.705	-0.828	-2.06	<1E-04
8251	298757	Hs.312485	Complement factor I	CFI	0.007	-1.187	-0.01	<1E-04
8264	131377	Hs.518123	TRK-fused gene	TFG	-0.428	-0.071	6.03	<1E-04
8266	198208	Hs.476319	Enoyl Coenzyme A hydratase domain containing 2	ECHDC2	-0.137	-0.322	0.43	<1E-04
8284	197421	In multiple clusters	In multiple clusters		0.491	-0.263	-1.87	<1E-04
8293	204339	Hs.256022	RAR-related orphan receptor C	RORC	0.605	-0.409	-1.48	<1E-04
8317	25718	Hs.435556	In multiple clusters		0.583	0.146	3.99	<1E-04
8318	42365	Hs.513043	IMP3, U3 small nucleolar ribonucleoprotein, homolog	IMP3	0.409	-0.549	-0.74	<1E-04
8336	131376	Hs.239666	Core 1 synthase, glycoprotein-N-acetylgalactosamine 1	C1GALT1	0.525	-0.515	-1.02	<1E-04
8345	135976	Hs.24719	Modulator of apoptosis 1	MOAP1	0.259	-0.187	-1.39	<1E-04
8372	131779	Hs.77961	In multiple clusters		0.363	-0.339	-1.07	<1E-04
8411	236207	Hs.406134	Data not found		0.133	-0.129	-1.03	<1E-04
8417	135187	Hs.504003	Beta-site APP-cleaving enzyme 1	BACE1	0.485	-0.514	-0.94	<1E-04
8421	275796	Hs.444213	Transducin-like enhancer of split 4	TLE4	0.416	-0.128	-3.25	<1E-04
8440	308473	Hs.439552	Eukaryotic translation elongation factor 1 alpha 1	EEF1A1	0.203	-0.935	-0.22	<1E-04
8490	47454	Hs.188757	Potassium channel tetramerisation domain containing 20	KCTD20	0.388	0.026	14.93	<1E-04
8494	309585	Hs.221127	Solute carrier family 39, member 11	SLC39A11	-0.178	-0.660	0.27	<1E-04
8501	263899	Hs.522584	Thymosin, beta 4, X-linked	TMSB4X	0.490	-0.614	-0.8	<1E-04
8503	320696	In multiple clusters	Trophoblast-derived noncoding RNA	TncRNA	1.046	-0.739	-1.42	<1E-04
8504	357775	Hs.438863	Nuclear receptor subfamily 1, group H, member 3	NR1H3	0.848	-0.777	-1.09	<1E-04
8521	320699	Hs.372286	Cullin 3	CUL3	-0.070	-0.629	0.11	<1E-04
8536	230049	Hs.506908	Arylacetamide deacetylase (esterase)	AADAC	0.111	-0.334	-0.33	<1E-04
8552	142791	Hs.524910	Ferritin, heavy polypeptide 1	FTH1	0.428	-0.394	-1.09	<1E-04
8561	140162	Hs.72071	Potassium channel tetramerisation domain containing 9	KCTD9	0.905	-0.064	-14.04	<1E-04
8566	309938	Hs.83169	Matrix metallopeptidase 1 (interstitial collagenase)	MMP1	0.462	-0.662	-0.7	<1E-04
8575	321906	In multiple clusters	Eukaryotic translation elongation factor 1 alpha 1	EEF1A1	0.526	-0.716	-0.73	<1E-04
8583	293879	Hs.470588	Karyopherin alpha 6 (importin alpha 7)	KPNA6	0.562	-0.440	-1.28	<1E-04
8584	310535	Hs.558335	Glutathione S-transferase theta 1	GSTT1	0.360	-0.568	-0.63	<1E-04
8587	38862	Hs.503709	Transmembrane protein 123	TMEM123	0.300	-0.396	-0.76	<1E-04

8593	320299	Hs.9527	Chromosome 2 open reading frame 28	C2orf28	0.236	-0.626	-0.38	<1E-04
8611	322241	In multiple clusters	In multiple clusters		0.446	-0.747	-0.6	<1E-04
8627	264214	Hs.408528	Retinoblastoma 1 (including osteosarcoma)	RB1	-0.019	-0.385	0.05	<1E-04
8633	139222	Hs.205163	Mitochondrial ribosomal protein L3	MRPL3	0.469	-0.080	-5.9	<1E-04
8656	305814	Hs.194718	Zinc finger, RAN-binding domain containing 2	ZRANB2	0.298	-0.633	-0.47	<1E-04
8674	301899	Hs.79110	Nucleolin	NCL	0.048	-0.748	-0.06	<1E-04
8692	301902	Hs.469944	CDNA FLJ23679 fis, clone HEP09084		0.272	-0.552	-0.49	<1E-04
8698	197760	Hs.356331	Peptidylprolyl isomerase A (cyclophilin A)	PPIA	0.156	-0.672	-0.23	<1E-04
8699	233550	Hs.505934	In multiple clusters		0.369	-0.282	-1.31	<1E-04
8701	300104	Hs.534168	NADH dehydrogenase 1 alpha subcomplex, 1, 7.5kDa	NDUFA1	0.595	-0.730	-0.81	<1E-04
8717	239703	Hs.436911	Alpha-1-microglobulin/bikunin precursor	AMBP	0.226	-0.499	-0.45	<1E-04
8726	246293	Hs.405880	Mitochondrial ribosomal protein S21	MRPS21	0.039	-0.596	-0.07	<1E-04
8746	306181	Hs.534125	Major histocompatibility complex, class I, B	HLA-B	0.598	-0.624	-0.96	<1E-04
8755	299604	Hs.432387	In multiple clusters		0.043	-0.640	-0.07	<1E-04
8765	341101	Hs.180877	In multiple clusters		0.580	-0.656	-0.88	<1E-04
8782	306538	Hs.558521	Chromosome 2 open reading frame 33	C2orf33	0.188	-0.548	-0.34	<1E-04
8825	241297	Hs.515016	Melanoma associated antigen (mutated) 1	MUM1	0.870	-0.244	-3.57	<1E-04
8849	134349	Hs.24485	Structural maintenance of chromosomes 3	SMC3	1.100	-0.197	-5.59	<1E-04
8852	244367	Hs.118118	Tetraspanin 5	TSPAN5	0.072	-0.363	-0.2	<1E-04
8858	142920	In multiple clusters	In multiple clusters		0.107	-1.046	-0.1	<1E-04
8873	345360	Hs.456465	In multiple clusters		0.443	-0.050	-8.81	<1E-04
8881	310755	Hs.502829	Splicing factor 1	SF1	0.685	-0.380	-1.8	<1E-04
8924	249650	Hs.35052	Testis enhanced gene transcript (BAX inhibitor 1)	TEGT	0.175	-0.570	-0.31	<1E-04
8943	292401	Data not found	MRNA; cDNA DKFZp781B2436		0.327	-0.692	-0.47	<1E-04
8971	321950	Hs.514581	Actin, gamma 1	ACTG1	1.093	-0.539	-2.03	<1E-04
8978	249366	Hs.78466	Proteasome 26S subunit, non-ATPase, 8	PSMD8	-0.107	-0.506	0.21	<1E-04
9024	296601	Hs.504641	CD163 molecule	CD163	0.203	-0.745	-0.27	<1E-04
9031	208987	Hs.370668	SEC14 and spectrin domains 1	SESTD1	-0.143	0.230	-0.62	<1E-04
9042	298089	Hs.29978	Frataxin	FXN	0.300	-0.239	-1.26	<1E-04
9047	140063	Hs.520210	Full-length cDNA clone CS0DC014YA20		0.193	-0.411	-0.47	<1E-04

9051	291549	Hs.432424	Tripeptidyl peptidase II	TPP2	0.449	-0.107	-4.22	<1E-04
9052	308915	Hs.432485	Similar to large subunit ribosomal protein L36a	LOC729362	0.233	-0.337	-0.69	<1E-04
9065	139270	Hs.102471	Phosphatase and actin regulator 2	PHACTR2	1.820	0.242	7.51	<1E-04
9067	211847	Hs.171802	Ring finger protein 149	RNF149	1.784	0.668	2.67	<1E-04
9078	270128	Hs.260603	Phosphatidylinositol-4-phosphate 5-kinase, type II, beta	PIP5K2B	1.432	0.519	2.76	<1E-04
9080	325080	Hs.494691	Profilin 1	PFN1	0.625	-0.238	-2.63	<1E-04
9147	147590	Hs.238990	Cyclin-dependent kinase inhibitor 1B (p27, Kip1)	CDKN1B	0.393	-0.115	-3.41	<1E-04
9149	233241	Hs.124147	F-box and leucine-rich repeat protein 11	FBXL11	-0.021	-0.417	0.05	<1E-04
9204	269007	Hs.231029	Hypothetical protein MGC14327	MGC14327	0.111	-0.021	-5.27	<1E-04
9230	244774	Hs.471851	High density lipoprotein binding protein (vigilin)	HDLBP	-0.429	0.005	-82.6	<1E-04
9254	38025	Hs.369052	In multiple clusters		0.251	-0.687	-0.37	<1E-04
9263	135813	Hs.522632	TIMP metallopeptidase inhibitor 1	TIMP1	0.192	-0.812	-0.24	<1E-04
9268	301988	Hs.373763	Heterogeneous nuclear ribonucleoprotein R	HNRPR	0.040	-0.509	-0.08	<1E-04
9281	136462	Hs.533262	Anaphase promoting complex subunit 2	ANAPC2	0.280	-0.482	-0.58	<1E-04
9284	242806	Hs.513578	Adenylate cyclase 7	ADCY7	0.779	-0.422	-1.85	<1E-04
9299	140377	Hs.327736	Kinesin family member 5B	KIF5B	0.384	-0.039	-9.82	<1E-04
9304	308545	In multiple clusters	In multiple clusters		-0.021	-0.861	0.02	<1E-04
9321	292036	Hs.516837	Smg-5 homolog, nonsense mediated mRNA decay factor	SMG5	0.752	-0.227	-3.31	<1E-04
9335	137236	Hs.508010	Fibronectin type III domain containing 3A	FNDC3A	0.047	-0.403	-0.12	<1E-04
9365	262407	Hs.435771	Apoptosis inhibitor 5	API5	-0.001	-0.915	0	<1E-04
9384	295067	Hs.73965	Splicing factor, arginine/serine-rich 2	SFRS2	0.290	-0.471	-0.62	<1E-04
9385	322984	Hs.412842	Cell division cycle 123 homolog (S. cerevisiae)	CDC123	0.274	-0.373	-0.74	<1E-04
9392	253258	Hs.128556	Dentin matrix acidic phosphoprotein	DMP1	0.678	-0.655	-1.03	<1E-04
9401	262433	Hs.532411	Lysosomal trafficking regulator	LYST	0.400	-0.623	-0.64	<1E-04
9403	321230	Data not found	Major histocompatibility complex, class II, DR beta 1	HLA-DRB1	1.132	-0.518	-2.18	<1E-04
9420	297010	In multiple clusters	In multiple clusters		0.018	-0.778	-0.02	<1E-04
9421	320774	In multiple clusters	In multiple clusters		-0.084	-0.773	0.11	<1E-04
9430	308901	Hs.90061	Progesterone receptor membrane component 1	PGRMC1	0.111	-0.421	-0.26	<1E-04
9479	139888	In multiple clusters	Enoyl Coenzyme A hydratase domain containing 1	ECHDC1	-0.171	-0.686	0.25	<1E-04
9484	307365	Hs.512934	General transcription factor IIA, 2, 12kDa	GTF2A2	0.080	-0.284	-0.28	<1E-04

9488	145727	Hs.464071	Phosphogluconate dehydrogenase	PGD	0.099	-0.371	-0.27	<1E-04
9497	139169	Hs.536655	CDNA clone IMAGE:5299642		1.237	-0.150	-8.24	<1E-04
9512	338477	Hs.501140	KIAA1598	KIAA1598	0.369	-0.138	-2.67	<1E-04
9525	149334	Hs.28491	Spermidine/spermine N1-acetyltransferase 1	SAT1	0.006	-0.722	-0.01	<1E-04
9574	301614	Hs.37558	Riboflavin kinase	RFK	0.774	-0.579	-1.34	<1E-04
9591	290622	Hs.461379	GABA(A) receptor-associated protein-like 2	GABARAPL[illegible]	0.081	-0.853	-0.09	<1E-04
9608	245549	Hs.282331	Sirtuin 5 (S. cerevisiae)	SIRT5	0.033	-0.620	-0.05	<1E-04
9610	301642	Hs.479396	Full length insert cDNA clone YY93G09		0.139	-0.556	-0.25	<1E-04
9617	240846	Hs.520640	Full-length cDNA clone CS0DF034YA13		0.371	-0.541	-0.69	<1E-04
9637	301429	Hs.20716	Translocase of inner mitochondrial membrane 17A	TIMM17A	0.891	-0.266	-3.35	<1E-04
9646	305873	Hs.534255	Beta-2-microglobulin	B2M	1.021	-0.901	-1.13	<1E-04
9671	233274	Hs.64691	F-box protein 28	FBXO28	2.996	1.219	2.46	<1E-04
9672	270863	Hs.369453	defective in cullin neddylation 1, domain containing 2	DCUN1D2	-0.228	0.078	-2.94	<1E-04
9682	302012	In multiple clusters	Major histocompatibility complex, class I, A	HLA-A	0.494	-0.331	-1.49	<1E-04
9705	146879	Hs.519601	, dominant negative helix-loop-helix protein	ID4	1.314	0.593	2.22	<1E-04
9724	229809	Hs.404056	Data not found		0.422	0.078	5.4	<1E-04
9753	293640	Hs.132225	Phosphoinositide-3-kinase, regulatory subunit 1	PIK3R1	0.935	-0.719	-1.3	<1E-04
9762	297048	Hs.68714	Splicing factor, arginine/serine-rich 1	SFRS1	0.345	-0.645	-0.54	<1E-04
9763	321652	Hs.355983	Basic leucine zipper and W2 domains 1	BZW1	0.632	-0.611	-1.03	<1E-04
9771	292802	Hs.520898	Cathepsin B	CTSB	0.488	-0.966	-0.5	<1E-04
9772	310464	Hs.522110	CAMP responsive element binding protein 3	CREB3	1.804	0.687	2.63	<1E-04
9778	219847	Hs.522493	Calmodulin regulated spectrin-associated protein 1	CAMSAP1	1.467	0.129	11.33	<1E-04
9781	321590	Hs.499891	Heterogeneous nuclear ribonucleoprotein H3 (2H9)	HNRPH3	0.727	-0.679	-1.07	<1E-04
9784	34349	Hs.356285	High-mobility group nucleosome binding domain 1	HMGN1	0.359	-0.410	-0.88	<1E-04
9814	214066	Hs.324746	Alpha-2-HS-glycoprotein	AHSG	0.067	-0.355	-0.19	<1E-04
9817	320842	Hs.314359	Eukaryotic translation initiation factor 3, subunit 12	EIF3S12	0.119	-0.532	-0.22	<1E-04
9824	246706	Hs.374596	Tumor protein, translationally-controlled 1	TPT1	0.284	-0.741	-0.38	<1E-04
9826	309203	Hs.474010	Pituitary tumor-transforming 1 interacting protein	PTTG1IP	0.335	-0.723	-0.46	<1E-04
9842	248244	In multiple clusters	In multiple clusters		0.321	-0.343	-0.94	<1E-04
9853	320847	In multiple clusters	In multiple clusters		1.496	-0.138	-10.81	<1E-04

9863	346544	Hs.204044	Data not found		0.301	-0.153	-1.96	<1E-04
9907	322771	Hs.78524	Prune homolog (Drosophila)	PRUNE	0.550	-0.404	-1.36	<1E-04
9942	266328	Hs.505104	Kelch domain containing 5	KLHDC5	0.696	-0.158	-4.41	<1E-04
9960	266353	Hs.78989	Alcohol dehydrogenase 5 (class III), chi polypeptide	ADH5	0.173	-0.401	-0.43	<1E-04
9998	323166	Hs.464472	Myosin regulatory light chain MRLC2	MRLC2	0.211	-0.631	-0.33	<1E-04
10022	245978	Hs.172685	Exportin 7	XPO7	0.186	-0.472	-0.39	<1E-04
10033	301106	Hs.347524	Chromosome 16 open reading frame 75	C16orf75	0.366	-0.333	-1.1	<1E-04
10042	305895	Hs.180171	CDNA FLJ10417 fis, clone NT2RP1000112		0.621	-0.258	-2.41	<1E-04
10101	148403	Hs.398636	In multiple clusters		0.303	0.018	16.71	<1E-04
10119	149132	Hs.518326	In multiple clusters		0.383	-0.262	-1.46	<1E-04
10137	146926	Hs.433300	Fc fragment of IgE, receptor for; γ polypeptide	FCER1G	0.769	-0.244	-3.15	<1E-04
10148	245614	Hs.181301	Cathepsin S	CTSS	0.642	-0.188	-3.42	<1E-04
10149	271726	Hs.200668	Ceramide kinase	CERK	0.841	0.158	5.33	<1E-04
10167	291288	Hs.503251	In multiple clusters		1.313	-0.050	-26.42	<1E-04
10175	264558	In multiple clusters	Lactate dehydrogenase B	LDHB	0.397	-0.840	-0.47	<1E-04
10176	297050	Hs.458986	Data not found		1.852	-0.096	-19.3	<1E-04
10185	294218	Hs.224171	Enolase 3 (beta, muscle)	ENO3	0.515	-0.552	-0.93	<1E-04
10193	262146	In multiple clusters	In multiple clusters		0.232	-0.753	-0.31	<1E-04
10199	136924	Hs.1063	Small nuclear ribonucleoprotein polypeptide C	SNRPC	0.221	-0.377	-0.59	<1E-04
10200	48070	Hs.269528	N-acetyltransferase 13	NAT13	0.119	-0.245	-0.49	<1E-04
10254	48162	Hs.289293	Chromosome 8 open reading frame 42	C8orf42	1.481	-0.322	-4.6	<1E-04
10265	258685	Hs.271643	LMBR1 domain containing 1	LMBRD1	0.210	-0.309	-0.68	<1E-04
10313	347371	In multiple clusters	Ribosomal protein L41	RPL41	0.024	-0.825	-0.03	<1E-04
10333	37303	Hs.473761	Reticulon 3	RTN3	-0.556	0.083	-6.69	<1E-04
10344	48411	Hs.446271	Hypothetical gene supported by AK091718	LOC401504	0.743	0.184	4.04	<1E-04
10362	48413	Hs.386294	Zinc finger protein 195	ZNF195	2.369	2.722	0.87	<1E-04
10378	416017	Hs.509523	Data not found		-0.419	0.200	-2.09	<1E-04
10414	376584	Hs.108080	Cysteine and glycine-rich protein 1	CSRP1	-0.678	-0.033	20.73	<1E-04
10428	1673750	Hs.469879	Data not found		2.040	0.263	7.75	<1E-04
10435	754835	Hs.98367	SRY (sex determining region Y)-box 17	SOX17	2.278	1.427	1.6	<1E-04

10464	1673907	Hs.46319	Sex hormone-binding globulin	SHBG	1.724	0.092	18.67	<1E-04
10473	125145	Hs.163079	Tubulin, alpha-like 3	TUBAL3	1.227	0.230	5.33	<1E-04
10526	788248	Hs.28309	In multiple clusters		-0.648	0.084	-7.69	<1E-04
10556	234920	In multiple clusters	In multiple clusters		-1.675	-0.058	29.13	<1E-04
10583	1436996	Data not found	Data not found		-1.154	1.367	-0.84	<1E-04
10594	416976	Hs.145384	Glycine-N-acyltransferase	GLYAT	-0.673	-0.047	14.29	<1E-04
10603	452306	Hs.511367	Data not found		-0.664	0.071	-9.4	<1E-04
10615	757922	Hs.532626	Chromosome 14 open reading frame 179	C14orf179	0.281	-0.355	-0.79	<1E-04
10618	1714994	Hs.523036	PQ loop repeat containing 2	PQLC2	-0.472	0.006	-85.31	<1E-04
10619	2229759	Hs.88139	Phosphoribosylformylglycinamidine synthase	PFAS	-0.944	-0.113	8.34	<1E-04
10642	774069	Hs.129634	Cyclin-dependent kinase 2-interacting protein	CINP	0.345	-0.125	-2.75	<1E-04
10662	249150	Hs.16064	CNKSR family member 3	CNKSR3	-0.258	0.368	-0.7	<1E-04
10673	2260357	Hs.415336	Transcribed locus		-1.477	0.072	-20.48	<1E-04
10698	247281	Hs.46468	Chemokine (C-C motif) receptor 6	CCR6	1.039	0.113	9.17	<1E-04
10725	192653	Hs.521973	WD repeat domain 13	WDR13	1.876	0.047	39.77	<1E-04
10734	253432	Hs.437831	Full length insert cDNA clone YW27A09		2.273	1.117	2.04	<1E-04
10763	2280621	Hs.80642	Signal transducer and activator of transcription 4	STAT4	-0.771	0.085	-9.03	<1E-04
10781	2286609	Hs.180402	Zinc finger protein 671	ZNF671	-0.998	-0.041	24.55	<1E-04
10826	292933	Hs.436912	Kinesin family member C1	KIFC1	-0.844	-0.292	2.89	<1E-04
10834	1258037	Hs.514885	Homo sapiens, clone IMAGE:4698842, mRNA		-0.724	-0.002	396.26	<1E-04
10883	485720	Hs.533122	Splicing factor, arginine/serine-rich 10	SFRS10	0.050	-0.616	-0.08	<1E-04
10905	123913	Hs.560937	Data not found		1.213	0.218	5.56	<1E-04
10907	1978918	Hs.117062	Apoptosis-inducing factor, mitochondrion-associated, 2	AIFM2	-0.687	0.157	-4.37	<1E-04
10914	1675423	Hs.530077	Paraoxonase 2	PON2	1.849	0.255	7.24	<1E-04
10933	358936	Hs.323445	In multiple clusters		-0.942	0.162	-5.83	<1E-04
10943	1987547	Hs.128841	CDNA FLJ41537 fis, clone BRTHA2017985		-0.785	0.039	-20.25	<1E-04
10951	489919	Hs.534359	In multiple clusters		-0.778	-0.070	11.19	<1E-04
10970	193713	Hs.496459	Transcribed locus		-0.478	0.156	-3.07	<1E-04
10979	1986767	Hs.7165	Zinc finger protein 259	ZNF259	-0.711	0.082	-8.65	<1E-04
10988	205986	Hs.538381	Transcribed locus		-0.613	-0.024	25.32	<1E-04

11006	198699	Hs.89714	Chemokine (C-X-C motif) ligand 5	CXCL5	-0.733	-0.033	22.27	<1E-04
11015	1989328	Hs.44898	CDNA FLJ40901 fis, clone UTERU2003704		-0.838	0.095	-8.84	<1E-04
11059	2334538	Hs.432281	Transcribed locus		-0.447	0.111	-4.04	<1E-04
11067	204295	Hs.559465	Data not found		-0.894	0.022	-40.93	<1E-04
11096	83458	Hs.201667	Aldo-keto reductase family 1, member D1	AKR1D1	-1.105	0.135	-8.16	<1E-04
11099	490305	Hs.482087	Leucine rich repeat containing 8 family, member D	LRRC8D	0.140	-0.478	-0.29	<1E-04
11112	233794	Hs.549405	Data not found		0.152	-0.739	-0.21	<1E-04
11167	2163031	Hs.550714	CDNA FLJ37358 fis, clone BRAMY2023172		-1.013	-0.032	31.74	<1E-04
11194	1641931	Hs.474083	UDP-Gal:betaGlcNAc beta 1,4- galactosyltransferase	B4GALT2	-0.794	0.228	-3.49	<1E-04
11204	86245	Hs.525557	Serpin peptidase inhibitor, clade A, member 1	SERPINA1	-0.959	-0.161	5.95	<1E-04
11240	294582	In multiple clusters	In multiple clusters		-0.992	-0.029	34.31	<1E-04
11244	704619	Hs.523680	Structure specific recognition protein 1	SSRP1	-0.273	0.108	-2.52	<1E-04
11258	229505	Hs.556017	Chromosome 1 open reading frame 131	C1orf131	-1.191	-0.247	4.82	<1E-04
11266	954219	Data not found	Data not found		-0.727	0.085	-8.54	<1E-04
11276	1269464	Hs.518129	Transcribed locus		-1.147	0.150	-7.67	<1E-04
11285	1987401	Hs.310449	Solute carrier family 23, member 1	SLC23A1	-1.018	0.031	-32.35	<1E-04
11290	731118	Hs.128846	Signal-regulatory protein alpha	SIRPA	1.776	1.016	1.75	<1E-04
11294	446159	Hs.100426	Breast cancer metastasis suppressor 1	BRMS1	-1.303	-0.120	10.87	<1E-04
11310	1848406	Hs.111944	Cytochrome P450, family 3, subfamily A, polypeptide 7	CYP3A7	2.230	0.327	6.82	<1E-04
11328	1848515	Hs.431550	Data not found		-0.579	0.296	-1.96	<1E-04
11348	430171	Hs.211038	Chromosome 8 open reading frame 31	C8orf31	-1.134	-0.106	10.71	<1E-04
11382	1848643	Hs.527119	Data not found		1.883	0.556	3.39	<1E-04
11393	2005398	Hs.9641	Complement component 1, q subcomponent, A chain	C1QA	-0.876	-0.011	79.37	<1E-04
11410	1524020	Hs.143674	RAS guanyl releasing protein 3	RASGRP3	-1.049	0.056	-18.76	<1E-04
11419	725736	Hs.23960	Cyclin B1	CCNB1	-0.782	0.134	-5.86	<1E-04
11420	1084278	Hs.1247	Transcribed locus		-1.691	0.013	-134.71	<1E-04
11428	1518787	Hs.405942	Coiled-coil domain containing 137	CCDC137	-0.649	0.358	-1.81	<1E-04
11456	109209	Data not found	ATP-binding cassette, sub-family D (ALD), member 3	ABCD3	-0.164	0.111	-1.48	<1E-04
11464	1841283	Hs.501684	Nucleosome assembly protein 1-like 4	NAP1L4	-0.601	-0.029	20.44	<1E-04
11482	1714594	Hs.536848	Transcribed locus		-0.607	0.156	-3.88	<1E-04

11492	109804	Hs.546557	SFT2 domain containing 2	SFT2D2	-1.090	0.000	-7057.62	<1E-04
11499	51120	Hs.107510	Phosphatidic acid phosphatase type 2 domain containing 2	PPAPDC2	-0.683	0.109	-6.24	<1E-04
11546	110550	Data not found	Data not found		-0.742	0.113	-6.56	<1E-04
11553	210323	Hs.282703	Data not found		1.897	0.484	3.92	<1E-04
11555	2446486	Hs.502	Transporter 2, ATP-binding cassette, sub-family B	TAP2	-0.775	0.220	-3.52	<1E-04
11573	2442658	Hs.152337	Protein arginine methyltransferase 3	PRMT3	-1.026	0.067	-15.3	<1E-04
11582	111734	Data not found	Data not found		-0.369	0.171	-2.16	<1E-04
11608	1693357	Hs.1407	Endothelin 2	EDN2	-1.356	-0.018	76.93	<1E-04
11636	113551	Hs.518505	Large subunit GTPase 1 homolog (S. cerevisiae)	LSG1	-0.746	0.136	-5.47	<1E-04
11645	2445047	Hs.468653	KIAA1841	KIAA1841	-1.127	-0.077	14.7	<1E-04
11660	811031	Hs.304682	Cystatin C (amyloid angiopathy and cerebral hemorrhage)	CST3	-0.639	0.443	-1.44	<1E-04
11699	2141529	Hs.83077	Interleukin 18 (interferon-gamma-inducing factor)	IL18	-0.730	-0.303	2.41	<1E-04
11706	1850476	Hs.461263	Data not found		-0.595	0.074	-8.06	<1E-04
11717	2143952	Hs.146591	B lymphoid tyrosine kinase	BLK	-0.974	0.064	-15.24	<1E-04
11744	81761	Data not found	Data not found		-1.139	0.098	-11.67	<1E-04
11798	85164	Hs.119983	Mannan-binding lectin serine peptidase 2	MASP2	-0.919	0.160	-5.75	<1E-04
11833	320922	Hs.553498	Transcribed locus		-1.128	0.142	-7.94	<1E-04
11843	2154486	Hs.386567	Transcribed locus		-1.333	0.121	-11.05	<1E-04
11851	305302	Hs.478682	Coiled-coil domain containing 50	CCDC50	-0.647	0.135	-4.79	<1E-04
11861	2156749	Hs.167700	SMAD family member 5	SMAD5	-0.958	0.117	-8.18	<1E-04
11870	85642	Data not found	Data not found		-0.771	0.302	-2.55	<1E-04
11875	753071	Hs.484885	Pleckstrin homology-like domain, family A, member 1	PHLDA1	-0.361	0.255	-1.42	<1E-04
11879	2177567	Hs.105103	Pellino homolog 2 (Drosophila)	PELI2	-1.506	0.157	-9.62	<1E-04
11906	115880	Hs.559340	Hypothetical protein LOC285074	LOC285074	-1.096	0.010	-108.81	<1E-04
11936	502305	Hs.114309	Apolipoprotein L, 1	APOL1	1.692	0.421	4.02	<1E-04
11959	2393153	Hs.73952	Proline-rich protein HaeIII subfamily 1	PRH1	-0.439	0.188	-2.33	<1E-04
11960	123516	Hs.325404	Phenylalanine hydroxylase	PAH	-0.746	0.221	-3.37	<1E-04
11978	123857	Hs.112143	Data not found		-0.930	0.104	-8.91	<1E-04
11979	108405	Hs.439552	Eukaryotic translation elongation factor 1 alpha 1	EEF1A1	0.275	-0.689	-0.4	<1E-04
11996	124611	Hs.94011	Necdin-like 2	NDNL2	-1.117	0.145	-7.69	<1E-04

12032	126718	Hs.273077	Transmembrane protein 14B	TMEM14B	-0.937	0.106	-8.84	<1E-04
12040	1674302	Hs.135399	Transcribed locus		-0.873	0.045	-19.39	<1E-04
12049	2405473	Hs.210044	Transcribed locus		-0.831	0.327	-2.54	<1E-04
12050	129329	Hs.3887	Data not found		-0.933	0.503	-1.86	<1E-04
12058	1901687	Hs.486489	Ectonucleotide pyrophosphatase/phosphodiesterase 3	ENPP3	-1.232	0.119	-10.34	<1E-04
12068	30236	Data not found	Adrenergic, beta, receptor kinase 2	ADRBK2	-0.543	0.540	-1.01	<1E-04
12077	2451368	Hs.6834	Formin binding protein 4	FNBP4	-0.764	0.158	-4.82	<1E-04
12086	41581	Hs.516316	semaphorin 6C	SEMA6C	-0.723	0.569	-1.27	<1E-04
12095	2457201	Hs.445482	Transcribed locus		-1.022	-0.025	41.65	<1E-04
12097	359905	Hs.43666	Protein tyrosine phosphatase type IVA, member 3	PTP4A3	1.772	0.609	2.91	<1E-04
12122	85438	Hs.511872	Cytochrome P450, family 2, subfamily C, polypeptide 18	CYP2C18	-1.058	0.087	-12.18	<1E-04
12123	321906	In multiple clusters	Eukaryotic translation elongation factor 1 alpha 1	EEF1A1	0.272	-0.797	-0.34	<1E-04
12200	809860	Hs.129159	Chromosome 7 open reading frame 11	C7orf11	0.955	0.048	19.84	<1E-04
12201	127242	Hs.52931	Adrenergic, alpha-1A-, receptor	ADRA1A	2.165	0.502	4.32	<1E-04
12220	1031919	Hs.244940	Retinol dehydrogenase 10 (all-trans)	RDH10	-1.032	0.142	-7.28	<1E-04
12224	470126	Hs.433381	Chromosome 6 open reading frame 89	C6orf89	1.264	1.127	1.12	<1E-04
12229	738939	Hs.133444	Excision repair cross-complementing	ERCC6	-1.014	0.077	-13.22	<1E-04
12232	375662	Hs.386939	Ubiquitin specific peptidase 7 (herpes virus-associated)	USP7	1.823	0.410	4.45	<1E-04
12269	487370	Hs.501794	ATPase type 13A1	ATP13A1	0.559	-0.093	-5.99	<1E-04
12274	1618665	Hs.416553	CDNA FLJ36415 fis, clone THYMU2010917		-0.838	0.121	-6.94	<1E-04
12283	786574	Hs.508010	Fibronectin type III domain containing 3A	FNDC3A	-0.157	0.237	-0.66	<1E-04
12286	375668	Hs.183994	In multiple clusters		-0.485	0.009	-55.18	<1E-04
12293	2186619	Hs.125742	Leucine-rich repeats and calponin homology domain	LRCH4	-0.701	0.171	-4.1	<1E-04
12298	742679	Hs.503074	Transmembrane protein 16A	TMEM16A	-0.639	0.377	-1.7	<1E-04
12311	2186859	Hs.376208	Lymphotoxin beta (TNF superfamily, member 3)	LTB	-0.903	0.081	-11.19	<1E-04
12428	155415	Hs.388024	Zinc finger protein 76 (expressed in testis)	ZNF76	-0.695	0.456	-1.53	<1E-04
12445	2368650	Hs.439155	Data not found		-1.053	0.055	-19.12	<1E-04
12472	1650110	Hs.558398	Sine oculis homeobox homolog 1 (Drosophila)	SIX1	-0.941	0.130	-7.26	<1E-04
12481	2255057	Hs.2303	Erythropoietin	EPO	-0.576	0.135	-4.28	<1E-04
12490	1734951	Hs.72901	Cyclin-dependent kinase inhibitor 2B (p15, inhibits CDK4)	CDKN2B	-0.817	0.173	-4.73	<1E-04

12499	2321583	Hs.96704	Full length insert cDNA clone ZA96G04		-1.521	0.021	-73.6	<1E-04
12508	1732823	Hs.143873	S100 calcium binding protein A10	S100A10	-0.865	0.207	-4.18	<1E-04
12517	2467712	Hs.400625	Glutamate-rich WD repeat containing 1	GRWD1	-0.455	0.374	-1.22	<1E-04
12555	308473	Hs.439552	Eukaryotic translation elongation factor 1 alpha 1	EEF1A1	0.272	-0.817	-0.33	<1E-04
12586	742711	Hs.517106	In multiple clusters		0.612	-0.270	-2.27	<1E-04
12652	1187874	Hs.473847	SH3 domain binding glutamic acid-rich protein	SH3BGR	-1.198	-0.031	38.06	<1E-04
12673	365727	In multiple clusters	In multiple clusters		0.139	-0.674	-0.21	<1E-04
12688	898255	Hs.386684	Abelson helper integration site 1	AHI1	-1.095	-0.008	136.48	<1E-04
12706	942520	Hs.187624	Hypothetical protein FLJ20674	FLJ20674	-1.057	0.024	-43.99	<1E-04
12715	301919	Hs.129563	Chromosome 17 open reading frame 59	C17orf59	-1.217	-0.082	14.91	<1E-04
12721	756490	Hs.512670	Branched chain aminotransferase 2, mitochondrial	BCAT2	-0.811	0.318	-2.55	<1E-04
12724	1589998	Hs.465558	Non-metastatic cells 6	NME6	-0.627	0.160	-3.91	<1E-04
12725	2208194	Hs.461722	Trafficking protein particle complex 2-like	TRAPPC2L	-0.857	0.119	-7.19	<1E-04
12733	781339	In multiple clusters	In multiple clusters		-0.491	0.290	-1.69	<1E-04
12771	302012	In multiple clusters	Major histocompatibility complex, class I, A	HLA-A	0.140	-0.560	-0.25	<1E-04
12843	202916	In multiple clusters	In multiple clusters		0.259	-0.435	-0.6	<1E-04
12901	760039	Hs.42217	Integrin alpha FG-GAP repeat containing 1	ITFG1	-0.945	-0.137	6.88	<1E-04
12904	1682514	Hs.124854	Chromosome 7 open reading frame 13	C7orf13	-1.196	0.020	-60.15	<1E-04
12913	2305614	Hs.407004	Transcribed locus		-0.687	0.175	-3.92	<1E-04
12914	221223	Hs.164060	Data not found		-1.242	-0.245	5.07	<1E-04
12922	1723169	Hs.194061	Ets variant gene 2	ETV2	-1.178	0.162	-7.27	<1E-04
12931	2069130	Hs.476636	Membrane associated guanylate kinase	MAGI1	-1.093	0.087	-12.57	<1E-04
12987	110210	Hs.355643	RNA binding protein S1, serine-rich domain	RNPS1	0.099	-0.385	-0.26	<1E-04
12989	486650	In multiple clusters	In multiple clusters		0.611	-0.807	-0.76	<1E-04
13024	376134	Hs.76152	Aquaporin 1 (Colton blood group)	AQP1	-0.406	0.208	-1.96	<1E-04
13079	485964	Hs.91531	Myeloid/lymphoid or mixed-lineage leukemia	MLLT6	-0.089	-0.426	0.21	<1E-04
13102	1390584	Hs.507498	Mitochondrial intermediate peptidase	MIPEP	-0.418	0.168	-2.49	<1E-04
13120	1638302	Hs.49421	WD repeat domain 78	WDR78	-0.836	0.077	-10.85	<1E-04
13129	774107	Hs.134859	V-maf musculoaponeurotic fibrosarcoma oncogene homolog	MAF	-0.472	0.055	-8.64	<1E-04
13151	487102	In multiple clusters	In multiple clusters		0.134	-0.769	-0.17	<1E-04

13157	2219539	Hs.10319	UDP glucuronosyltransferase 2 family, polypeptide B7	UGT2B7	-1.212	0.070	-17.27	<1E-04
13207	758211	Hs.131212	Zinc finger and BTB domain containing 39	ZBTB39	1.627	0.265	6.13	<1E-04
13256	247761	Data not found	Data not found		-0.670	0.322	-2.08	<1E-04
13310	249067	Hs.518355	G elongation factor, mitochondrial 1	GFM1	-0.428	0.412	-1.04	<1E-04
13328	260177	Hs.435326	Actin-like 6A	ACTL6A	-0.582	0.144	-4.04	<1E-04
13342	773531	Hs.417022	Intestinal cell (MAK-like) kinase	ICK	-0.850	-0.081	10.54	<1E-04
13346	270523	Hs.203691	Data not found		-0.712	0.092	-7.72	<1E-04
13360	781142	In multiple clusters	In multiple clusters		1.613	1.202	1.34	<1E-04
13362	275395	Hs.13205	Beta 1,3-galactosyltransferase-like	B3GALTL	-0.083	0.533	-0.16	<1E-04
13427	2222735	Hs.107410	Glycine N-methyltransferase	GNMT	-0.386	0.281	-1.37	<1E-04
13462	898040	Hs.189174	Doublesex and mab-3 related transcription factor 3	DMRT3	-1.243	0.130	-9.55	<1E-04
13475	486009	Hs.11169	ERBB receptor feedback inhibitor 1	ERRFI1	-0.022	-1.261	0.02	<1E-04
13519	366897	In multiple clusters	Ribosomal protein S17	RPS17	-0.216	-0.443	0.49	<1E-04
13537	365824	Hs.23650	MYC-associated zinc finger protein	MAZ	0.514	-0.408	-1.26	<1E-04
13540	740224	In multiple clusters	DALR anticodon binding domain containing 3	DALRD3	1.174	0.055	21.24	<1E-04
13553	2224715	Hs.121520	Adhesion molecule with Ig-like domain 2	AMIGO2	-0.763	0.115	-6.62	<1E-04
13561	726744	Hs.493887	Exosome component 3	EXOSC3	-0.722	0.390	-1.85	<1E-04
13570	1636365	Hs.129614	Transmembrane protein 27	TMEM27	-0.717	0.076	-9.45	<1E-04
13579	377693	Hs.476320	Small nucleolar RNA host gene (non-protein coding) 8	SNHG8	-0.178	0.349	-0.51	<1E-04
13583	486020	Hs.99936	Keratin 10	KRT10	0.673	-0.256	-2.63	<1E-04
13592	471738	Hs.175120	Hypothetical protein FLJ11286	FLJ11286	0.493	-0.050	-9.93	<1E-04
13630	769010	Hs.124553	Zinc finger protein 263	ZNF263	1.448	1.151	1.26	<1E-04
13640	813536	Hs.464971	Phosphoinositide-3-kinase, class 3	PIK3C3	1.978	0.348	5.69	<1E-04
13678	1680246	Hs.1955	Transcribed locus		-0.770	-0.011	70.15	<1E-04
13696	1736295	Hs.479954	Methylenetetrahydrofolate dehydrogenase 2-like	MTHFD2L	-0.811	0.228	-3.57	<1E-04
13720	772455	Hs.534338	In multiple clusters		1.733	0.309	5.61	<1E-04
13768	1755833	Hs.471162	Ras association and pleckstrin homology domains 1	RAPH1	-0.274	0.310	-0.89	<1E-04
13777	2414066	Hs.488181	Transcribed locus		-0.216	0.307	-0.7	<1E-04
13793	1662419	Hs.348297	Chromosome 20 open reading frame 82	C20orf82	1.264	0.345	3.66	<1E-04
13795	2115733	Hs.438794	Data not found		-0.505	0.299	-1.69	<1E-04

13799	488685	Hs.332382	BTB (POZ) domain containing 10	BTBD10	0.383	-0.696	-0.55	<1E-04
