# Supplementary material for: Early and long-standing rheumatoid arthritis: distinct molecular signatures identified by gene-expression profiling in synovia
Source: Arthritis Res Ther. 2009 Jun 29;11(3):R99. doi: 10.1186/ar2744 (PMC2714155; doi:10.1186/ar2744)
Supplement: Additional data file 3 — Word file containing a table listing the ontological classes of differentially expressed genes in early RA versus long-standing RA. [file ar2744-S3.doc]

**Table S3** Ontological classes of differentially expressed genes in early RA *versus* long-standing RA

Gene regulation Ontological classes *P* value

Biological process Up Immunity and defense 0.002

Proteolysis 0.003

Protein metabolism and modification 0.003

MHCII-mediated immunity 0.004

Tumor suppressor 0.02

Oncogenesis 0.02

Pentose-phosphate shunt 0.02

T-cell–mediated immunity 0.02

mRNA splicing 0.03

Ectoderm development 0.03

Other homeostasis activities 0.04

Stress response 0.04

Down Cell surface-receptor–mediated signal transduction 0.001

Cell cycle 0.001

Cell cycle control 0.003

Ligand-mediated signaling 0.005

Mitochondrial transport 0.01

Reverse transcription 0.02

Oogenesis 0.03

Signal transduction 0.04

Other cell cycle process 0.04

Intracellular signaling cascade 0.04

Granulocyte-mediated immunity 0.05

Inhibition of apoptosis 0.05

Molecular function Up Serine/threonine protein kinase receptor 0.004

Major histocompatibility complex antigen 0.005

Protease inhibitor 0.005

Cyclase 0.009

Adenylate cyclase 0.01

Voltage-gated potassium channel 0.02

Other chaperones 0.02

Other actin family cytoskeletal protein 0.02

Molecular function Up Defense/immunity protein 0.02

Lyase 0.03

mRNA processing factor 0.03

Signaling molecule 0.04

Voltage-gated ion channel 0.04

Serine protease inhibitor 0.04

mRNA splicing factor 0.05

HMG box transcription factor 0.05

Down Select regulatory molecule 0.004

Hydrolase 0.01

Cytokine 0.02

Reverse transcriptase 0.02

Kinase modulator 0.03

Protease inhibitor 0.03

Other kinase 0.04

Signaling molecule 0.04

Peptide hormone 0.05

Pathways Up Blood coagulation 0.0007

Hypoxia response via HIF activation 0.003

Plasminogen activating cascade 0.01

Ionotropic glutamate receptor pathway 0.01

T cell activation 0.01

Heterotrimeric G-protein signaling pathway G_i_-alpha– and G_s_-alpha–mediated pathway 0.04

Endothelin signaling pathway 0.05

Down EGF receptor signaling pathway 0.01

Hypoxia response via HIF activation 0.02

Valine biosynthesis 0.02

Leucine biosynthesis 0.02

Isoleucine biosynthesis 0.02

Cysteine biosynthesis 0.02

Alanine biosynthesis 0.02

p53 pathway 0.02

Axon guidance mediated by netrin 0.02

Endothelin signaling pathway 0.03
