# Supplementary material for: Early and long-standing rheumatoid arthritis: distinct molecular signatures identified by gene-expression profiling in synovia
Source: Arthritis Res Ther. 2009 Jun 29;11(3):R99. doi: 10.1186/ar2744 (PMC2714155; doi:10.1186/ar2744)
Supplement: Additional data file 4 — Adobe file containing a table listing the 116 genes differentially expressed at early RA versus controls. [file ar2744-S4.pdf]

Table S4 List of 116 genes differentially expressed at early RA (eRA) *versus* Controls (C)

uniqID	IMAGE Id	UGCluster	Name	Symbol	eRA Group mean	C Group mean	Fold change	P value
269	194954	Hs.151411	Transcribed locus		0.586	0.007	88.765	<1E-04
337	115506	Hs.337766	Ribosomal protein L18a	RPL18A	1.334	-0.050	-26.883	<1E-04
409	115772	Data not found	Data not found		0.456	-0.062	-7.373	<1E-04
793	84418	Hs.238996	Transcribed locus		0.493	-0.113	-4.360	<1E-04
1099	84101	Hs.318894	G protein-coupled receptor 126	GPR126	0.460	-0.006	-75.176	<1E-04
1126	81796	Hs.1360	Cytochrome P450, family 2, subfamily B6	CYP2B6	0.386	-0.047	-8.207	<1E-04
1155	113265	Hs.355708	Transcribed locus		0.479	-0.028	-17.139	<1E-04
1163	109718	Data not found	Data not found		0.478	-0.137	-3.492	<1E-04
1549	83383	Hs.469593	Data not found		0.423	-0.109	-3.894	<1E-04
1604	110243	Hs.567242	Data not found		2.733	-0.001	-2083.262	<1E-04
1630	83045	Data not found	Data not found		0.611	-0.052	-11.810	<1E-04
1663	37354	Hs.188757	Potassium channel tetramerisation domain	KCTD20	0.378	-0.068	-5.554	<1E-04
1677	113347	Hs.111903	Fc fragment of IgG, receptor, transporter, alpha	FCGRT	0.489	-0.081	-6.068	<1E-04
1715	122131	Hs.246112	Activating signal cointegrator 1 complex	ASCC3L1	0.362	0.008	44.839	<1E-04
1756	78051	Hs.154078	Lipopolysaccharide binding protein	LBP	0.547	-0.093	-5.860	<1E-04
1794	111825	Hs.443417	Misshapen-like kinase 1 (zebrafish)	MINK1	0.515	-0.006	-87.636	<1E-04
1876	113779	Hs.472492	Placenta-specific 4	PLAC4	0.368	-0.189	-1.945	<1E-04
1948	116040	Hs.431550	Mitogen-activated protein kinase kinase kinase kinase 4	MAP4K4	-0.900	0.236	-3.810	<1E-04
2060	197077	Hs.408909	Golgi phosphoprotein 3 (coat-protein)	GOLPH3	0.548	-0.131	-4.176	<1E-04
2155	115318	Hs.563417	CDNA FLJ45287 fis, clone BRHIP3002124		0.244	-0.159	-1.538	<1E-04
2224	78956	Hs.567289	Data not found		0.494	0.012	40.370	<1E-04
2380	116522	Hs.272168	Serine incorporator 3	SERINC3	-0.367	-0.070	5.233	<1E-04
2438	196350	Hs.482103	Transcribed locus		0.478	-0.078	-6.099	<1E-04
2690	191801	Data not found	Data not found		0.801	-0.074	-10.875	<1E-04
2962	83074	Data not found	Data not found		0.492	-0.042	-11.727	<1E-04
2969	195547	Hs.136829	Regulatory factor X, 3	RFX3	0.504	-0.018	-28.725	<1E-04
3071	109302	Data not found	Data not found		0.342	-0.023	-15.012	<1E-04
3077	193757	Hs.119825	Spectrin, alpha, erythrocytic 1 (elliptocytosis 2)	SPTA1	0.322	-0.040	-8.065	<1E-04

3293	196032	Hs.434973	Glycophorin A (MNS blood group)	GYPA	0.513	-0.060	-8.551	<1E-04
3302	196827	Hs.546286	Ribosomal protein S3	RPS3	0.599	-0.015	-38.776	<1E-04
3311	195299	Hs.504877	Rho GDP dissociation inhibitor (GDI) beta	ARHGDIB	0.296	-0.118	-2.498	<1E-04
3405	113539	Data not found	Data not found		0.438	-0.032	-13.698	<1E-04
3425	123106	Hs.434961	Ataxin 1	ATXN1	0.502	-0.117	-4.306	<1E-04
3462	346009	In multiple clusters	Data not found		-1.926	-0.117	16.499	<1E-04
3550	241837	Hs.552968	Data not found		0.462	-0.137	-3.380	<1E-04
3711	234985	Hs.53454	Topoisomerase (DNA) II binding protein 1	TOPBP1	0.498	0.032	15.789	<1E-04
3999	212698	Hs.409794	C-type lectin domain family 1, member B	CLEC1B	0.465	-0.073	-6.367	<1E-04
4015	198503	Hs.474584	Transcribed locus		0.223	-0.231	-0.964	<1E-04
4161	240009	Hs.356794	Ribosomal protein S24	RPS24	0.436	-0.133	-3.269	<1E-04
4182	417033	Hs.34780	Doublecortex; lissencephaly, X-linked (doublecortin)	DCX	-1.053	-0.080	13.172	<1E-04
4440	230056	Data not found	Data not found		0.529	-0.038	-13.827	<1E-04
4867	417468	Hs.59134	Transmembrane protein 188	TMEM188	0.328	-0.067	-4.901	<1E-04
5079	235116	Data not found	Data not found		0.588	-0.065	-9.093	<1E-04
5167	202414	Hs.221498	Data not found		0.342	-0.039	-8.800	<1E-04
5187	213585	Hs.521124	Mitochondrial ribosomal protein S28	MRPS28	0.510	-0.251	-2.034	<1E-04
5336	22328	Hs.512914	NMDA receptor regulated 1-like	NARG1L	0.775	0.052	15.040	<1E-04
5345	114544	Hs.14601	Hematopoietic cell-specific Lyn substrate 1	HCLS1	0.399	-0.102	-3.921	<1E-04
5500	200938	Hs.158209	Data not found		0.505	0.017	30.457	<1E-04
5501	207970	Data not found	Data not found		0.838	-0.149	-5.620	<1E-04
5626	200604	Hs.268787	Ariadne homolog	ARIH1	0.531	-0.035	-15.138	<1E-04
5696	110385	Hs.512619	Thymidine kinase 2, mitochondrial	TK2	0.255	-0.148	-1.722	<1E-04
5829	415630	Hs.387794	Transcribed locus		-0.314	-0.034	9.342	<1E-04
5878	201365	Data not found	Data not found		0.339	0.032	10.520	<1E-04
5887	202531	Hs.212088	Epoxide hydrolase 2, cytoplasmic	EPHX2	0.651	-0.059	-10.946	<1E-04
5955	415621	Hs.71465	Squalene epoxidase	SQLE	-0.594	-0.045	13.243	<1E-04
6265	202704	Hs.109655	Sex comb on midleg-like 1 (Drosophila)	SCML1	0.640	0.015	43.595	<1E-04
6515	113522	Hs.484551	Hypothetical protein MGC5370	MGC5370	0.747	0.028	26.659	<1E-04
6542	23108	Hs.270775	Transcribed locus		0.564	-0.072	-7.840	<1E-04

6572	203767	Data not found	Data not found		0.306	-0.095	-3.231	<1E-04
6820	430313	Hs.379414	Malignant fibrous histiocytoma amplified sequence 1	MFHAS1	1.644	-0.046	-36.096	<1E-04
6979	200743	Hs.80756	Betaine-homocysteine methyltransferase	BHMT	0.623	-0.718	-0.867	<1E-04
7073	343218	Hs.509791	Enhancer of rudimentary homolog (Drosophila)	ERH	0.243	-0.345	-0.703	<1E-04
7495	299084	Hs.308709	Data not found		0.601	-0.132	-4.543	<1E-04
8042	248066	Data not found	Data not found		-0.847	-0.002	342.433	<1E-04
8264	131377	Hs.518123	TRK-fused gene	TFG	-0.428	-0.018	23.714	<1E-04
8503	320696	In multiple clusters	Data not found		1.046	-0.125	-8.388	<1E-04
8504	357775	Hs.438863	Nuclear receptor subfamily 1, group H, member 3	NR1H3	0.848	-0.141	-6.000	<1E-04
8905	211829	Hs.556795	Data not found		0.381	-0.008	-46.755	<1E-04
10344	48411	Hs.446271	Hypothetical gene supported by AK091718	LOC401504	0.743	0.005	149.733	<1E-04
10378	416017	Hs.509523	Menage a trois homolog 1, cyclin H assembly factor	MNAT1	-0.419	0.114	-3.688	<1E-04
10619	2229759	Hs.88139	Data not found		-0.944	-0.030	31.150	<1E-04
10642	774069	Hs.129634	Cyclin-dependent kinase 2-interacting protein	CINP	0.345	-0.266	-1.294	<1E-04
10709	2262577	Hs.69851	Nucleolar protein family A, member 1	NOLA1	-3.505	-0.280	12.512	<1E-04
10905	123913	Hs.560937	Data not found		1.213	-0.154	-7.869	<1E-04
10943	1987547	Hs.128841	CDNA FLJ41537 fis, clone BRTHA2017985		-0.785	0.033	-23.750	<1E-04
10951	489919	Hs.534359	Data not found		-0.778	-0.194	4.006	<1E-04
11006	198699	Hs.89714	Chemokine (C-X-C motif) ligand 5	CXCL5	-0.733	-0.074	9.956	<1E-04
11015	1989328	Hs.44898	Transcribed locus		-0.838	-0.025	33.339	<1E-04
11067	204295	Hs.559465	Transcribed locus		-0.894	0.018	-50.272	<1E-04
11096	83458	Hs.201667	Aldo-keto reductase family 1, member D1	AKR1D1	-1.105	-0.023	48.638	<1E-04
11203	2404052	Data not found	Data not found		-1.073	-0.083	12.909	<1E-04
11222	108369	Data not found	Data not found		-0.992	-0.064	15.573	<1E-04
11276	1269464	Hs.518129	Data not found		-1.147	-0.104	10.993	<1E-04
11294	446159	Hs.100426	Breast cancer metastasis suppressor 1	BRMS1	-1.303	-0.088	14.840	<1E-04
11393	2005398	Hs.9641	Data not found		-0.876	-0.058	15.121	<1E-04
11447	2140125	Hs.422662	Vaccinia related kinase 1	VRK1	-1.365	-0.069	19.858	<1E-04
11482	1714594	Hs.536848	Data not found		-0.607	0.002	-387.698	<1E-04
11546	110550	Data not found	Data not found		-0.742	-0.008	94.090	<1E-04

11660	811031	Hs.304682	Cystatin C	CST3	-0.639	-0.010	66.582	<1E-04
11812	741544	Hs.520026	Valyl-tRNA synthetase	VARS	1.952	-0.032	-60.714	<1E-04
11843	2154486	Hs.386567	Guanylate binding protein 2, interferon-inducible	GBP2	-1.333	0.051	-25.886	<1E-04
12095	2457201	Hs.445482	Transcribed locus		-1.022	-0.087	11.691	<1E-04
12229	738939	Hs.133444	CDNA FLJ13497 fis, clone PLACE1004518		-1.014	-0.065	15.570	<1E-04
12257	2188489	Hs.193268	Methylthioadenosine phosphorylase	MTAP	-1.274	-0.062	20.464	<1E-04
12275	2186985	Hs.489118	Sterile alpha motif domain containing 9-like	SAMD9L	-1.193	0.050	-24.002	<1E-04
12311	2186859	Hs.376208	Lymphotoxin beta (TNF superfamily, member 3)	LTB	-0.903	-0.002	442.956	<1E-04
12337	2119039	Hs.491869	Transcribed locus		-0.629	0.037	-16.774	<1E-04
12364	1690348	Hs.494552	Data not found		-0.349	-0.026	13.357	<1E-04
12481	2255057	Hs.2303	Erythropoietin	EPO	-0.576	-0.081	7.134	<1E-04
12508	1732823	Hs.143873	S100 calcium binding protein A10	S100A10	-0.865	0.015	-56.091	<1E-04
12586	742711	Hs.517106	CCAAT/enhancer binding protein (C/EBP), beta	CEBPB	0.612	-0.626	-0.977	<1E-04
12688	898255	Hs.386684	Abelson helper integration site 1	AHI1	-1.095	-0.059	18.498	<1E-04
12706	942520	Hs.187624	Transcribed locus		-1.057	-0.050	21.118	<1E-04
12715	301919	Hs.129563	Chromosome 17 open reading frame 59	C17orf59	-1.217	-0.119	10.217	<1E-04
12725	2208194	Hs.461722	Trafficking protein particle complex 2-like	TRAPPC2L	-0.857	0.035	-24.167	<1E-04
12901	760039	Hs.42217	Integrin alpha FG-GAP repeat containing 1	ITFG1	-0.945	-0.065	14.438	<1E-04
12904	1682514	Hs.124854	Data not found		-1.196	-0.109	10.995	<1E-04
12913	2305614	Hs.407004	Data not found		-0.687	-0.017	39.595	<1E-04
12914	221223	Hs.164060	Growth factor receptor-bound protein 10	GRB10	-1.242	-0.116	10.679	<1E-04
12931	2069130	Hs.476636	Transcribed locus		-1.093	-0.163	6.693	<1E-04
12934	428281	Hs.505601	SEC14-like 3 (S. cerevisiae)	SEC14L3	-0.627	0.013	-46.538	<1E-04
13102	1390584	Hs.507498	Mitochondrial intermediate peptidase	MIPEP	-0.418	0.005	-88.376	<1E-04
13157	2219539	Hs.10319	CDNA FLJ37609 fis, clone BRCOC2011010		-1.212	0.007	-171.278	<1E-04
13427	2222735	Hs.107410	Transcribed locus		-0.386	0.116	-3.336	<1E-04
13696	1736295	Hs.479954	Methylenetetrahydrofolate dehydrogenase 2-like	MTHFD2L	-0.811	-0.073	11.118	<1E-04
13791	726405	Hs.510635	Immunoglobulin heavy constant mu	IGHG1	2.049	-0.020	-104.834	<1E-04
