# Supplementary material for: Early and long-standing rheumatoid arthritis: distinct molecular signatures identified by gene-expression profiling in synovia
Source: Arthritis Res Ther. 2009 Jun 29;11(3):R99. doi: 10.1186/ar2744 (PMC2714155; doi:10.1186/ar2744)
Supplement: Additional data file 5 — Adobe file containing a table listing the 52 genes differentially expressed at long-standing RA versus controls. [file ar2744-S5.pdf]

Table S5 List of 52 genes differentially expressed in Long-standing RA (LSRA) *versus* Controls (C)

uniqID	IMAGE Id	UGCluster	Name	Symbol	LS RA Group mean	C Group mean	Fold change	P value
33	123991	Hs.524910	Ferritin, heavy polypeptide 1	FTH1	-0.985	-0.144	6.85	1.01E-03
42	122764	Hs.33642	Archain 1	ARCN1	-0.705	-0.102	6.90	1.18E-03
103	114323	Data not found			-1.319	-0.275	4.79	3.08E-03
111	110892	Hs.533732	Signal recognition particle 14kDa	SRP14	-0.872	-0.136	6.41	3.87E-03
309	113173	Data not found			0.360	-0.096	-3.76	9.25E-03
362	110105	Hs.701982	Interleukin 1 receptor, type I	IL1R1	0.204	-0.010	-21.11	2.13E-03
623	109097	Hs.397609	Ribosomal protein S16	RPS16	-0.794	-0.165	4.81	9.46E-03
1351	61466	Hs.292265	Zinc finger, MYND domain containing 11	ZMYND11	-0.839	0.115	-7.29	9.91E-03
1373	120298	Hs.469473	Ribosomal protein L31	RPL31	-0.469	-0.017	27.65	4.92E-03
1564	129473	Data not found			-0.864	-0.008	114.92	8.99E-03
1687	115389	Hs.433057	F-box and leucine-rich repeat protein 7	FBXL7	-0.554	0.053	-10.36	8.80E-03
1757	109205	Hs.381061	Ribosomal protein L19	RPL19	-0.966	-0.112	8.63	7.64E-03
2380	116522	Hs.272168	Serine incorporator 3	SERINC3	-0.289	-0.070	4.12	6.37E-03
2524	116104	Data not found			-1.356	-0.171	7.93	6.33E-04
3027	111193	Hs.381219	Ribosomal protein L15	RPL15	-0.461	0.017	-27.08	4.86E-03
4237	72927	Hs.286226	Myosin IC	MYO1C	0.685	0.121	5.65	5.13E-03
4309	21542	Hs.635441	Insulin-like growth factor binding protein 5	IGFBP5	-0.843	-0.037	22.99	1.87E-03
4475	207054	Hs.388664	Ribosomal protein L11	RPL11	-1.004	-0.163	6.16	7.16E-03
4830	347570	In multiple clusters			-0.931	-0.093	10.02	7.26E-03
4981	245474	Hs.361463	Coagulation factor X	F10	0.302	-0.071	-4.26	6.03E-03
5043	234320	Data not found			0.230	-0.206	-1.11	5.92E-03
5696	110385	Hs.512619	Thymidine kinase 2, mitochondrial	TK2	0.282	-0.148	-1.91	2.22E-03
5768	25214	In multiple clusters			0.347	-0.128	-2.71	8.40E-03
5924	211296	Hs.687315	Transcribed locus		-0.443	0.008	-57.11	6.75E-03
5947	78468	Hs.567508	Unc-93 homolog A (C. elegans)	UNC93A	0.513	0.005	99.63	5.41E-03
6096	229446	Data not found			0.383	0.0005	782.90	7.12E-03
6320	211423	Data not found			-0.665	-0.033	20.08	6.65E-04
6493	243312	Hs.42009	Transcribed locus		-0.488	-0.024	20.65	4.68E-03

6518	205115	Data not found			-0.596	-0.086	6.96	7.67E-03
6593	275950	Hs.624092	Transcribed locus		0.340	-0.087	-3.92	8.66E-03
6682	242945	In multiple clusters			0.510	-0.071	-7.15	1.55E-04
6725	208969	Hs.334846	Poliovirus receptor-related 1	PVRL1	-0.666	-0.043	15.33	5.46E-04
6761	207992	Hs.444959	Acyl-Coenzyme A oxidase 2, branched chain	ACOX2	-1.041	-0.271	3.84	6.63E-03
6834	233942	Data not found			0.353	-0.072	-4.91	4.79E-03
7113	149287	Hs.687966	Transcribed locus		0.439	0.050	8.74	7.42E-03
7585	310610	Hs.567235	Similar to 40S ribosomal protein S26	RPS26	-0.925	-0.089	10.38	7.92E-03
7647	293363	Hs.368592	Sortilin-related receptor	SORL1	-0.844	-0.074	11.36	7.56E-03
7922	42354	Hs.591916	cytochrome c oxidase assembly protein	COX15	-0.489	-0.035	13.90	7.61E-04
8288	327594	Hs.416024	Neurensin 2	NRSN2	-0.542	-0.009	63.65	6.47E-03
8510	251584	Hs.700368	Ubiquitin-conjugating enzyme E2H	UBE2H	-0.599	-0.123	4.85	6.58E-03
9263	135813	Hs.522632	TIMP metallopeptidase inhibitor 1	TIMP1	-1.011	-0.088	11.47	3.19E-04
9479	139888	Hs.486410	Enoyl Coenzyme A hydratase domain containing 1	ECHDC1	-0.738	-0.079	9.33	7.18E-03
9689	241175	Hs.149367	KIAA0776	KIAA0776	-1.359	-0.218	6.24	4.91E-04
9707	240856	Hs.50223	Retinol binding protein 4, plasma	RBP4	-0.534	-0.048	11.11	8.55E-03
9717	273214	Hs.529618	Transferrin receptor (p90, CD71)	TFRC	0.463	0.007	64.05	9.05E-03
10688	814358	Hs.710399	Transcribed locus		0.478	-0.077	-6.19	1.21E-03
10750	774178	Hs.436564	Prolyl endopeptidase	PREP	0.554	-0.064	-8.64	2.97E-03
11531	488838	Hs.522805	CD99 molecule-like 2	CD99L2	-0.834	-0.211	3.95	6.12E-03
11690	1405525	Hs.448520	Solute carrier family 7, member 2	SLC7A2	-1.268	-0.024	53.84	2.72E-04
12325	759192	Hs.658789	ADP-ribosylation factor-like 16	ARL16	0.524	-0.094	-5.58	5.80E-03
12792	714064	Hs.436657	Clusterin	CLU	-1.553	-0.086	17.99	6.70E-03
13359	725204	Hs.185674	Zinc finger protein 331	ZNF331	0.497	0.099	5.01	4.19E-03
